# Synthetic and semi-synthetic antioxidants in medicine and food industry: a review

**DOI:** 10.3389/fphar.2025.1599816

**Published:** 2025-07-22

**Authors:** Jan Tauchen, Lukáš Huml, Michal Jurášek, Joe M. Regenstein, Fatih Ozogul

**Affiliations:** ^1^ Department of Food Science, Faculty of Agrobiology, Food, and Natural Resources, Czech University of Life Sciences Prague, Kamýcká, Czechia; ^2^ Department of Chemistry of Natural Compounds, University of Chemistry and Technology Prague, Technická, Czechia; ^3^ Department of Food Science, Cornell University, Ithaca, NY, United States; ^4^ Department of Seafood Processing Technology, Faculty of Fisheries, Çukurova University, Adana, Türkiye; ^5^ Biotechnology Research and Application Center, Cukurova University, Adana, Türkiye

**Keywords:** free radical scavenging, chelation therapy, oxidative stress, semi-synthetic antioxidants, synthetic antioxidants

## Abstract

Oxidative stress is recognized as both a causative and contributing factor in many human diseases. As a result, significant research has been devoted to the development of synthetic and semi-synthetic antioxidants (ATs). This review summarizes the therapeutic potential of synthetic ATs, explores their possible clinical applications, and highlights novel structural modifications aimed at improving their pharmacological properties. Additionally, it presents ideas for refining current antioxidant testing methodologies. Despite the ongoing research, the therapeutic efficacy of synthetic ATs remains ambiguous for several reasons. These include the following: therapeutic benefits resulting from non-antioxidant mechanisms, insufficient dosage to elicit an antioxidant effect, poor oral bioavailability, a narrow therapeutic index, or toxicity that precludes clinical use. Nevertheless, some compounds, such as ebselen, edaravone, MitoQ10, and potentially N-acetylcysteine, have shown promising results. However, further studies are needed to confirm their efficacy and clarify whether their therapeutic effects are truly mediated through antioxidant mechanisms. Dietary antioxidants have achieved relatively higher clinical success, although their toxicity has also led to the withdrawal of some agents. One emerging therapeutic strategy involves inhibition of NADPH oxidase (NOX) enzymatic activity, with compounds such as ebselen, S17834, and GKT137831 showing potential across various disease models. Efforts to enhance antioxidant properties through molecular modifications, using advanced technologies such as prodrug strategies, nanotechnology, polymer complexation, targeted delivery systems, or conversion into inhalable formulations, have yielded variable success. Still, confirming the clinical relevance of newly developed antioxidants will require a paradigm shift in the testing approaches. Future studies must better define the molecular context of antioxidant action, including the following: which biomolecules are being protected, the specific radical species targeted, the tissue and subcellular distribution of the antioxidant, and how levels of endogenous antioxidants and reactive oxygen species (ROS) change post-administration (e.g., within the mitochondria). Despite extensive research, only a few synthetic antioxidants, such as edaravone, are currently used in clinical practice. Currently, no new antioxidant drugs are expected to receive regulatory approval in the near future.

## 1 Introduction

Oxidative stress, i.e., overproduction of free radicals, is believed to be a significant predictor and/or a source of secondary pathologies in human disease. However, the role of oxidative stress has only been proven for some types of cancer, neurodegenerative disorders (e.g., Alzheimer’s, Parkinson’s, and Huntington’s disease), and conditions involving chronic inflammation (Halliwell, 2012; Islam et al., 2024). There is an ongoing debate for cardiovascular diseases (e.g., atherosclerosis) and eye disorders (Cheah and Halliwell, 2021). For other human diseases, including diabetes, the role of oxidative stress on the onset and secondary pathology is not that important (or at least the results are contradictory) (Seet et al., 2010; Monnier et al., 2011; Halliwell, 2024).

**FIGURE 1:**
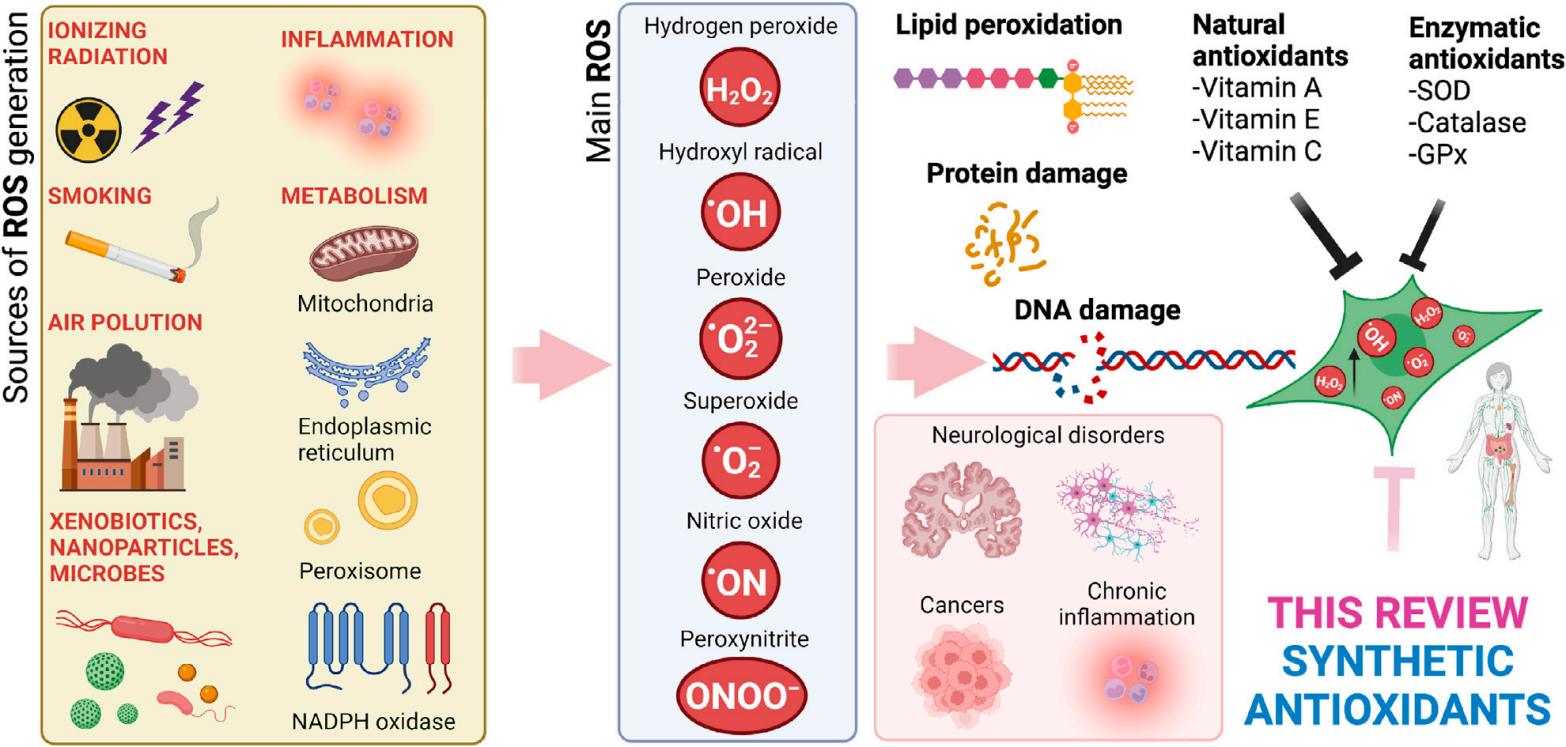
Image depicting sources of ROS/RNS (reactive nitrogen species), their formula, and their impact on human pathology. Note that ROS/RNS not only have deleterious effects, but they also have important physiological roles, including an immune response to bacteria and cancer cells and molecular signaling (Halliwell, 2024). Created with Biorender.com.

Free radicals are molecular entities containing at least one unpaired electron. Free radicals tend to be highly unstable and chemically reactive (however, this is not true for all free radicals). The biologically important free radicals include the oxygen-containing radical species (reactive species: RS, or reactive oxygen species: ROS). These include hydroxyl, superoxide and nitric oxide radicals, hydrogen peroxide, singlet oxygen, hypochlorous acid (HOCl), and peroxynitrite (Figure 1). They may damage important biomolecules, including lipids, proteins, and DNA (Lobo et al., 2010). However, free radicals also have indispensable physiological functions (e.g., acting as signaling molecules and regulating immune responses), and their deficiency may lead to health complications (Valko et al., 2007).

Although both animals and humans possess endogenous antioxidant defense systems, the prevailing view remains that additional antioxidant support must come from exogenous sources, particularly diet. However, many antioxidants, including so-called dietary antioxidants, have failed to demonstrate therapeutic benefits in human clinical trials (Halliwell, 2012; Halliwell et al., 2018). In some cases, they have even been associated with adverse effects, such as the increased risk of prostate cancer linked to vitamin E supplementation (Klein et al., 2011) (for more details, see Table 1). In recent years, the food and pharmaceutical industries have increasingly favored the use of natural products over synthetic compounds, based on the assumption that synthetic agents are inherently more toxic. However, this assumption is not always valid; some natural compounds such as benzoic and propionic acids can also exhibit toxicity (Shaw, 2018). From a therapeutic perspective, natural antioxidants may often be ineffective or even detrimental. Therefore, considerable attention is also being paid to synthetic antioxidants, which can be designed or modified to exert more favorable pharmacological and safety effects than their natural counterparts (see Table 2).

**TABLE 1:** Antioxidant activity of common ATs found in the human diet.

Compound	Mechanism of antioxidant action	Consequences of a deficiency	Comments	References
Vitamin E	Antioxidant activity using the phenolic -OH group that can donate H^•^ to peroxyl radicals. Thought to be useful in prevention of lipid peroxidation PUFA–O_2_^*^ + vitamin E–OH → PUFA–O_2_H + vitamin E–O^•^ However, vitamin E has failed to provide therapeutic benefits in many intervention studies and in some cases also produced toxic effects (e.g., prostate cancer)	Reproduction abnormalities, neurodegeneration, and erythrocyte hemolysis	Although being essential in human diet, its effects may not be entirely associated with AT mechanism. For example, vitamin E may act as a signaling molecule and regulate the expression of certain genes	Traber and Head (2021)
Vitamin C	Can scavenge several ROS, including O_2_^•-^, OH^•^, ROO^•^, and HOCl. Acts as a regeneration agent for vitamin E	Degradation of the collagen structure (e.g., scurvy)	Again, although being essential in human diet, its effects may not be entirely associated with an AT mechanism. It participates in synthesis of collagen, catecholamines, tocopherol, plastoquinones, and carnitine and activates hypothalamic hormones	Ursini et al. (2016)
Carotenoids	Can scavenge several ROS, especially singlet oxygen, and may protect human skin and eye from UV-induced oxidative damage	Not established, although β-carotene (and several others, but not all) acts as provitamin A in retinol synthesis	Since it participates in retinol synthesis, its effects may not be entirely associated with an AT mechanism	Halliwell and Gutteridge (2015)
Phenolic compounds	Can scavenge several ROS, including O_2_^−^ (some polyphenols only), ONOOH, and peroxyl radicals. However, many will act as pro-oxidants in the presence of transition metal ions. Failed in many clinical trials to provide a therapeutic benefit	Not established	Their bioavailability is very low with high metabolization and elimination rates. Many flavonoids will have other biological activities as well (e.g., anti-inflammatory). Their beneficial effects (if any) may not be entirely associated with an AT mechanism	Tauchen et al. (2020)
Ergothioneine	Can scavenge several ROS; has shown anti-inflammatory effects in some animal models	Not established, although it is absorbed from the diet and strongly retained in various tissues	Transported to tissues by specific transporter (OCTN1). Is deficient in certain diseases (e.g., Parkinson’s disease)	Cheah and Halliwell (2021)

**TABLE 2:** Possible advantages and disadvantages of natural *versus* semi-synthetic and synthetic ATs in medicinal applications.

Viewpoint	Natural AT	Semi-synthetic and synthetic ATs
Efficiency	Low to none (only very limited number of natural ATs are approved for medicinal use, e.g., silymarin (Ferenci, 2016)	Higher (in experimental conditions, they show better efficiency; however, even here only few examples are used in medicine, e.g., edaravone) (Halliwell, 2024)
Mode of action	If therapeutic benefits are observed, chances are that it is caused by a non-AT mechanism (e.g., anti-inflammatory action (flavonoids)) (Tauchen et al., 2020)	Therapeutic benefits are caused by a non-AT mechanism (e.g., anti-inflammatory action (edaravone)) (Halliwell, 2024)
Bioavailability	Very low; many natural ATs are found in nearly every higher plant (including dietary sources); animals (including humans) have evolved elimination and metabolizing mechanisms to limit the exposition to these compounds (e.g., flavonoids) (Tauchen et al., 2020)	Better; (semi)-synthetic ATs, in general, have better bioavailability (animal/human metabolism not used to them), and some can enter cells more readily, e.g., SOD mimetics (Day, 2008). However, many of them have low bioavailability as well
Oral availability	Orally available (some examples have poor oral availability, e.g., silymarin) (Di Costanzo and Angelico, 2019)	Generally good (although some derivatives require intravenous or other non-oral modes of administration, e.g., deferoxamine) (Yao et al., 2019)
Elimination half-life	Short (especially for flavonoids) (Manach and Donovan, 2004)	Generally longer (though, some analogs have a short half-life, e.g., thiols (Brock et al., 1984)
Blood–brain barrier (BBB) permeability	Low or negligible (though some, e.g., oxidized forms of vitamin C (Agus et al., 1997), were found to be able to cross the BBB)	Higher: some analogs were specifically developed to cross the BBB However, many examples have poor penetrating activity (e.g., many spin traps/nitroxides) (Halliwell and Gutteridge, 2015) or tirilazad mesylate (Cahill and Hall, 2017)
Targeting to specific tissues/organelles	Low to negligible (many lack the specific transportation system for targeted delivery, though few examples exist, e.g., ergothioneine and OCTN1 transporter) (Cheah and Halliwell, 2021)	Better (despite this, it was necessary to develop advanced technologies, e.g., nanomaterials, for many analogs to improve targeted delivery)
Toxicity	Generally low (though some are suggested to interfere with cytochrome P450-dependent enzymes; e.g., naringenin (Lu et al., 2011), and nordihydroguaiaretic acid shows renal and hepatic toxicity) (Manda et al., 2020)	Some analogs were developed to have lower toxicity. However, there are also many cases where the toxicity is quite profound (some may oxidize to form toxic products, e.g., thiols (Primas et al., 2023) or troglitazone) (Kassahun et al., 2001)
Stability	Low (e.g., many flavonoids are oxidized in solution, e.g., myricetin) (Cho et al., 2023)	Generally better (though some are prone to oxidation in solutions as well, e.g., *N*-acetylcysteine)

Generally, synthetic ATs appear to have more benefits over natural ones (indeed, they have been specifically developed to have improved properties compared to their natural counterparts). However, since only a few synthetic ATs have been approved for medicinal use thus far, it seems that their success in medicine is disappointing.

Synthetic AT may be divided into two main categories: (i) those that have been primarily developed and synthetized as AT and (ii) compounds that primarily act *via* different mechanisms of action, while their AT properties were discovered later, which may contribute to their biological activity (as observed for some clinically used drugs, e.g., 4-aminosalicylic acid with inflammatory bowel disease (IBD)). For any synthetic AT developed, it is important to know which biomolecule the agent is designed to protect, by which mechanism (scavenging RS, preventing RS formation, increasing endogenous defense mechanisms, and/or supporting oxidative damage repair), whether it can generate RS, and if there are any adverse effects associated with RS suppression. Many biologically active compounds whose benefits have been observed in clinical trials/animal studies might or might not exert antioxidant effects. It may be important to include measurements of biomarkers of oxidative damage (e.g., products of DNA and lipid oxidation, such as 8-hydroxy-2′-deoxyguanosine, 8-OHdG, or F_2_-isoprostanes) in observational studies, suggesting that the therapeutic benefit may actually be associated with an antioxidant effect (Moosmann and Behl, 2002; Halliwell and Gutteridge, 2015).

Many synthetic compounds with primary or secondary antioxidant activity have been developed. The aim of this review is to summarize the knowledge on synthetic AT. Since there are many ATs, including those that were not originally developed as ATs, some substances may have been inadvertently omitted. However, the most important examples of promising AT substances will be included, focusing on those for which clinical trials have been conducted. However, most synthetic ATs have not yet progressed to clinical trials. In these cases, animal and/or *in vitro* studies are considered. Unfortunately, they have many limitations, and their evidence is not nearly as high compared to that of clinical studies (this is further elaborated in the *Future Prospects* section). In some cases, interest in the substance has ended, and there is a lack of more recent studies—for this reason, an obsolete reference is used (because a newer one does not exist).

## 2 Methodology

The information for this review was obtained by performing a through literature review and search of relevant books and articles using the Web of Knowledge, SciVerse Scopus, and PubMed databases. Keyword searches were done using the names of different ATs (search period: 1960–2024; Figure 2). Chemical structures have been accurately depicted using ChemDraw software (ver 12.0.2; CambridgeSoft; Cambridge, USA). Some figures have been created with the help of BioRender image and illustration software (BioRender.com).

**FIGURE 2:**
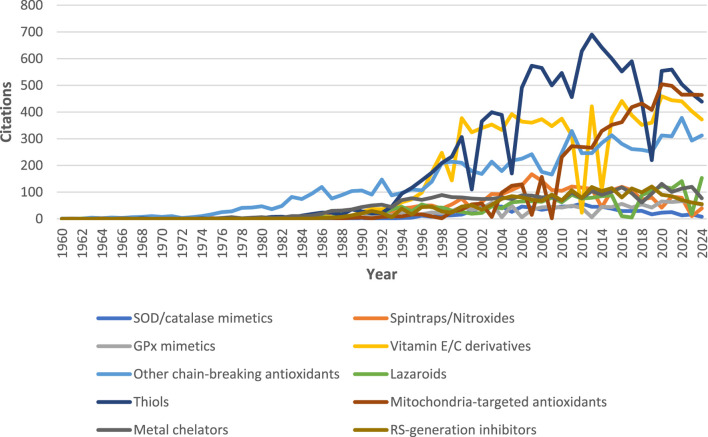
Number of articles containing the search keywords (compound names) in the title/abstract from 1960 to 2024 and merged into the corresponding categories. Data retrieved from PubMed on 3 December 2024. Note: the figure suggests that thiols (especially *N*-acetylcysteine, since it accounts for more than 50% of total number of citations on thiol), vitamin E/C derivatives, and related chain-breaking ATs are the most researched synthetic ATs. Mitochondria-targeted ATs are also attracting considerable research attention over the last decade.

## 3 Superoxide dismutase/catalase mimetics

**FIGURE 3:**
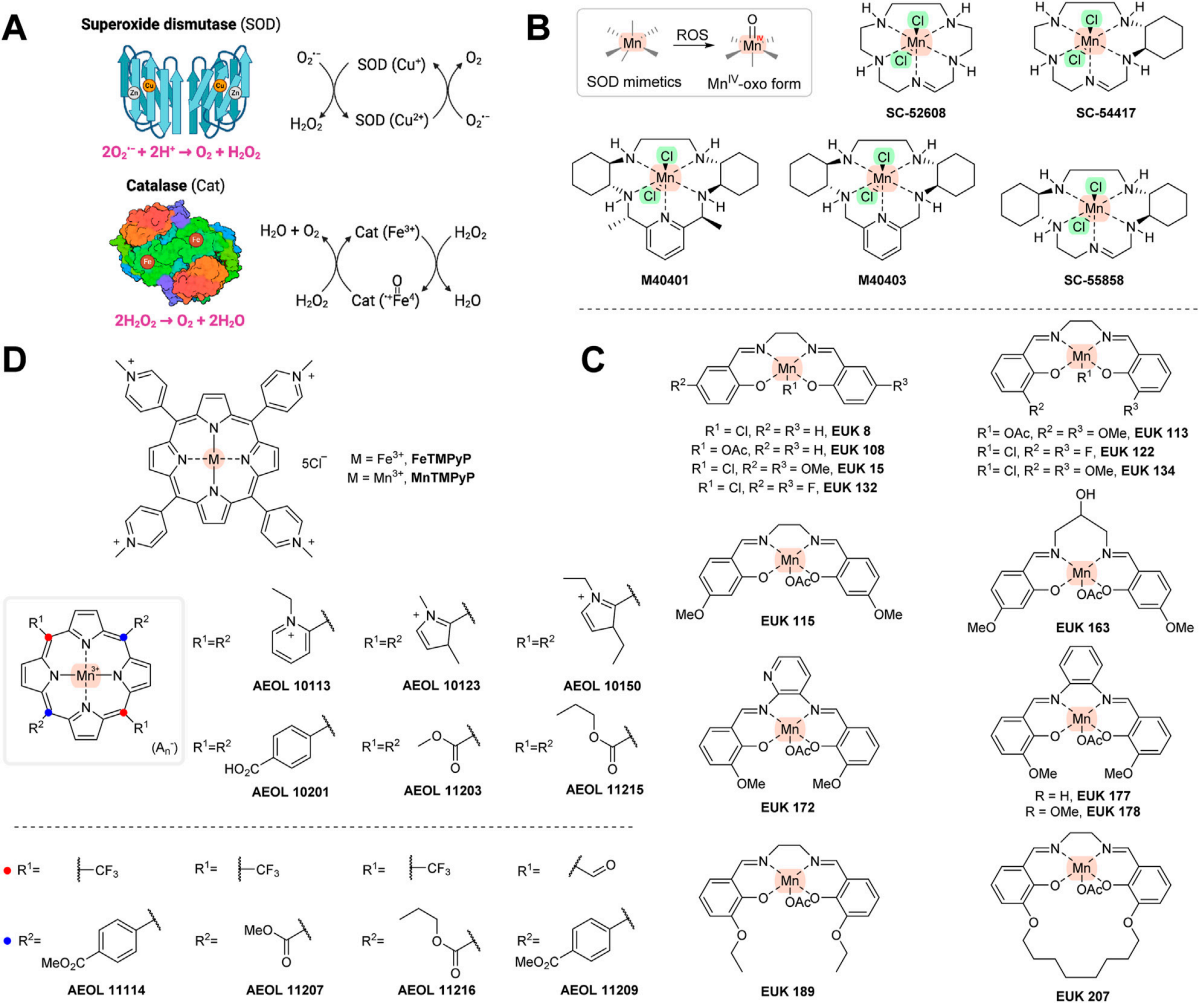
**(A)** Mechanism of superoxide dismutase and catalase. **(B)** One-electron-transferring manganese-containing SOD mimetics. **(C)** SOD/catalase mimetics of the EUK series. **(D)** Porphyrin-based SOD/catalase mimetics. Created with Biorender.com.

Superoxide dismutases (SOD; copper-, zinc-, or manganese-based) are part of the first line of AT defense in various biological systems, including humans (Figure 3; Table 3). A recombinant variant of SOD has been tested for therapeutic purposes, although it had limitations. The recombinant SOD (e.g., obtained from mice milk) has a short plasma half-life when injected into animals (Halliwell and Gutteridge, 2015). Therefore, conjugates with longer half-lives with varying therapeutic efficiency have been developed (Younus, 2018). Clinical trials with these recombinant SODs have been performed. For example, administration of recombinant CuZnSOD to premature infants attenuated inflammation, with subsequent improvement in clinical outcomes in later stages of life. Children treated with SOD had reduced incidences of pulmonary conditions (Li and Davis, 2003). Therefore, compounds mimicking the structure and activity of SOD as AT in the treatment of various diseases might give better results than recombinant variants. These are discussed below.

**TABLE 3:** Overview of endogenous AT and their proposed mechanisms of action.

Compound	Mechanism of antioxidant action	Comments	References
*Enzymic AT*			
Superoxide dismutases (SOD)	Dismutation of superoxide (2O_2_^•−^ *+* 2H → H_2_O_2_ + O_2_)	Multiple variants of SOD exist, including CuZnSOD and MnSOD. Recombinant SOD has been used in clinical practice; however, they have a short plasma half-life	Fridavich (1995)
Catalases	Degradation of peroxide (2H_2_O_2_ → 2H_2_O+ O_2_)	Especially helpful for high levels of H_2_O_2_	Hansberg (2022)
Glutathione peroxidases (GPx)	Removal of H_2_O_2_ with the use of reduced glutathione (2GSH + H_2_O_2_ → GSSG + 2H_2_O)	GPx assist peroxiredoxins to modulate H_2_O_2_ levels	Flohé et al. (2022)
Peroxiredoxins	Removal of peroxide with the use of thioredoxin [thioredoxin-(SH)_2_ + H_2_O_2_ → thioredoxin-(S)_2_ + 2H_2_O]	May also help remove lipid peroxides and peroxynitrite	Amponsah et al. (2021)
*Low-molecular weight AT*			
Glutathione (GSH)	Primarily acts as a substrate for GPx, but also was found to directly scavenge various RS, including OH^•^, ONOO^−^, and HOCl		Flohé et al. (2022)
Coenzyme Q_10_ (CoQ)	Essential electron carrier in the mitochondrial electron transport chain. Was also found to scavenge lipid peroxyl radicals. (PUFA–O_2_^•-^ + CoQH_2_ → PUFA–O_2_H + CoQH^•^)	CoQH_2_ may also recycle α-tocopherol radicals back to α-tocopherol	Bayır et al. (2020)
Melatonin	Primarily acts as a hormone controlling the circadian rhythm. Was found to potentially scavenge OH^•^		Monteiro et al. (2024)
Lipoic acid	Primarily a cofactor for various important enzymes. Is also able to regenerate GSH, vitamin C, and E		Zhang et al. (2017)
Uric acid	Final breakdown product of purine metabolism in primates. May provide antioxidant action especially in blood plasma, where its levels are high. (uric acid–O^-^ + R^•^ → uric acid–O^•^ + RH)		Kurajoh et al. (2021)

Some other endogenous molecules are sometimes regarded as AT as well, e.g., transferrin/lactoferrin, haptoglobin/hemopexin, albumin and ceruloplasmin. However, they act as passive AT, mainly in that they sequester transition metal ions, preventing RS formation.

SOD/catalase mimetics are low-molecular compounds that usually contain transition metals, i.e., manganese, iron, copper, or zinc. Their mechanism of action is similar to the naturally occurring counterparts they are designed to mimic, i.e., to neutralize superoxide radical (O_2_^•−^) and/or hydrogen peroxide (H_2_O_2_) and convert them to water (Figure 3A). SOD/catalase mimetics that are based on manganese and iron porphyrins unlike earlier copper derivatives might avoid high degrading activity and releasing copper ions *in vivo*, thus potentially providing a pro-oxidant effect. The metal centers of SOD/catalase mimetics are more open and can participate in redox reactions more readily with more different compounds than the original SOD/catalase enzymes. Additionally, as SOD/catalase mimetics are low-molecular weight compounds, they can enter cells more easily than their natural counterparts (Day, 2004; Halliwell and Gutteridge, 2015).

Some of the SOD/catalase mimetics, e.g., SC-52608, SC-55858 (Valdivia et al., 2009), SC-54417 (Doggrell, 2002), M40401, or M40403 (Shimizu et al., 2003) (Figure 3B), will react selectively with superoxide, but not with other RS (e.g., hydroxyl radicals, hydrogen peroxide, nitric oxide, or peroxynitrite). In these structures, manganese is held by five coordination bonds and is thus able to transfer just one electron. Although considered a specific superoxide scavenger, they are able to undergo one-electron transfers with other cellular redox-active agents and enzymes (including cytochrome P450 enzymes) (Day, 2004).

Non-selective manganese-based SOD mimetics capable of reacting with other free radicals have also been produced. Eukarion has developed a series of tetra-coordinated manganese structures (designated as EUK: e.g., EUK8, EUK134, EUK139, EUK189, and EUK207; Figure 3C) that were shown to react with superoxide, H_2_O_2,_ and peroxynitrite (Doctrow et al., 2002). The AEOL series (named after Aeolus Pharmaceuticals; Mission Viejo, California, USA; Figure 3D) contains a porphyrin system and scavenges these radicals as well (Day, 2004; Zhang et al., 2018; Forman and Zhang, 2021). Other related porphyrin ring-containing structures, such as FeTMPyP and MnTMPyP (Figure 3D), were shown to interfere with peroxynitirite. The AT mechanism of all of the above-mentioned compounds usually involves conversion of manganese to an Mn^IV^-oxo form, which is then reducible by endogenous or exogenous AT (e.g., glutathione or vitamin C) activity (Halliwell and Gutteridge, 2015). The SOD mimetics in animal models have benefits with a range of oxidative stress-related diseases, including inflammation, ischemia/reperfusion, shock, thrombosis, and diabetes. However, these compounds have thus far not had been subjected to clinical trials, possibly because of unwanted side effects, including lower Ca^2+^ transport or increased heme-oxygenase (HO-1) levels (Konorev et al., 2002).

In summary, since EUK series appear to be unique, in that they can scavenge both O_2_^•−^ and H_2_O_2_, they may be potentially more effective in conditions where the levels of both types of ROS are elevated (e.g., neurodegenerative diseases and ischemic injuries). The AEOL series and pentaazamacrocyclic ligand-based mimetics (SC series and M404 series), on the other hand, are more specialized toward O_2_^•−^ (though many can also address other ROS as well) and generally lack catalase-like activity (targeted to eliminate H_2_O_2_), and this may limit their therapeutic use (e.g., to more acute conditions such as lung injury or effects of ionizing radiation) (Day, 2008). However, AEOL series has shown to have better stability (are excreted unchanged in the urine) and oral bioavailability compared to the EUK series, which can suffer from toxicity and instability under certain conditions (Liang et al., 2007; 2021). The SC and M404 series also face solubility and bioavailability issues. All three series have shown varying degrees of efficacy in specific tissues, such as the lungs, brain, and cardiovascular system (Day, 2004). Enhancing their ability to reach specific tissues and sites of oxidative damage is an ongoing area of research. The proposed therapeutic benefit of SOD mimetics, including with animal models, has been summarized elsewhere (Halliwell and Gutteridge, 2015).

## 4 Spin traps/nitroxides

Spin traps are used to detect free radicals both *in vitro* and *in vivo*. The idea of using spin traps as therapeutic agents arose when α-phenyl-*tert*-butylnitrone (PBN) was shown to have protective effects in various animal models of ischemia–reperfusion (including intestinal, cardiac, and cerebral). Many spin traps react relatively ineffectively with superoxide (e.g., PBN has weak *in vitro* antioxidant activity), so high doses are required to achieve a therapeutic benefit. They accumulate rapidly in various tissues, although some spin traps/nitroxides have problems crossing the blood–brain barrier (BBB). On the other hand, many spin traps seem to be safe even in large doses. Their mode of action may not be directly related to an AT mechanism but may involve other effects, as some of them (including PBN) were shown to release NO (nitric oxide), which can inhibit ROS-producing enzymatic activity. PBN was also shown to interfere with genes encoding inducible nitric oxide synthase (iNOS), cyclooxygenase-2 (COX-2), and proinflammatory cytokines (Maples et al., 2004; Halliwell and Gutteridge, 2015).

In addition, PBN administration to old gerbils decreases the levels of brain protein carbonyls and improves cognitive functions. It can cross the BBB (concentrations in gerbil brain are estimated to approach 0.5 mM after an injection of 150 mg PBN/kg body weight (Yue et al., 1992). Several derivatives of PBN have been developed as potential therapeutic agents. CPI-1429 was shown to delay mortality as well as memory impairment in an aging mouse model (Floyd et al., 2002). After that, the interest appeared to cease. It is not known whether CPI-1429 is able to cross the BBB. However, the improved state of learning and improvement in memory deficits in the mice indicate the presence of some ability. NXY-059 (also known as Cerovive^®^) showed promising results in a primate model for stroke. It advanced to human clinical trials where it demonstrated effectiveness in the treatment of related ischemic injuries (Maples et al., 2004). However, it was not possible to obtain the initial results again, and this compound was also excluded from clinical trials (Antonic et al., 2018). NXY-059 is currently in human clinical trials for certain types of cancers (glioma) and auditory disorders (e.g., tinnitus and hearing loss). As for PBN, NXY-059 is a poor AT *in vitro*, so its activity may be due to other mechanisms of action (Maples et al., 2001). It is also hydrophilic, suggesting problems in transporting it across the BBB (unlike PBN) (Halliwell and Gutteridge, 2015). LPBNAH is another spin trap derivative. It ameliorated injury in isolated perfused rat heart (Tanguy et al., 2006) and increased the lifespan and neuroprotective action in a *Philodina acuticornis* (a species of freshwater bdelloid rotifers) model (Poeggeler et al., 2005). However, LPBNAH was more hydrophilic than NXY-059, so its ability to cross the BBB may be even worse. Stilbazulenyl nitrone (STAZN) showed neuroprotective effects in various animal models of ischemia/reperfusion (Belayev et al., 2002; Yang et al., 2005; Ley et al., 2007; 2008). Compared to NXY-059 and LPBNAH, STAZN is highly lipophilic and has been shown to cross the BBB (it reached a plasma concentration of ≈2.5% in the forebrain within 2–3 h after intravenous administration) (Ley et al., 2005).

The PBN molecule is converted to nitroxides with reactions with radicals. Nitroxides are radicals proposed as ATs. Examples are OXANO (Hasaniya et al., 2011), TEMPO (Yonekuta et al., 2007), and TEMPOL (Ciriminna and Pagliaro, 2010). As for PBN, the reaction with superoxide is quite slow (thus requiring large doses to be efficient *in vivo*) (Day, 2004). Nitroxides have been found to be capable of undergoing other redox reactions, including those involving peroxynitrite, carbonate radicals, and nitrogen dioxide. They can also interact with other entities, including vitamin C, Fe^2+^, NAD(P)H, and thiols, and they can inhibit myeloperoxidase activity. Since they show a range of redox reactions, their mechanism of action *in vivo* may not be related to an antioxidant effect. Some derivatives, e.g., 3-nitratomethylproxyl, in addition to typical nitroxide activity, can also donate NO. Available data suggest that these substances are capable of crossing the BBB (Zhang et al., 1998; Kwon et al., 2003). Some nitroxides (e.g., TEMPO) are used as stabilizers in plastics and polymers, and also as polymerization inhibitors (Moad et al., 2008).

**FIGURE 4:**
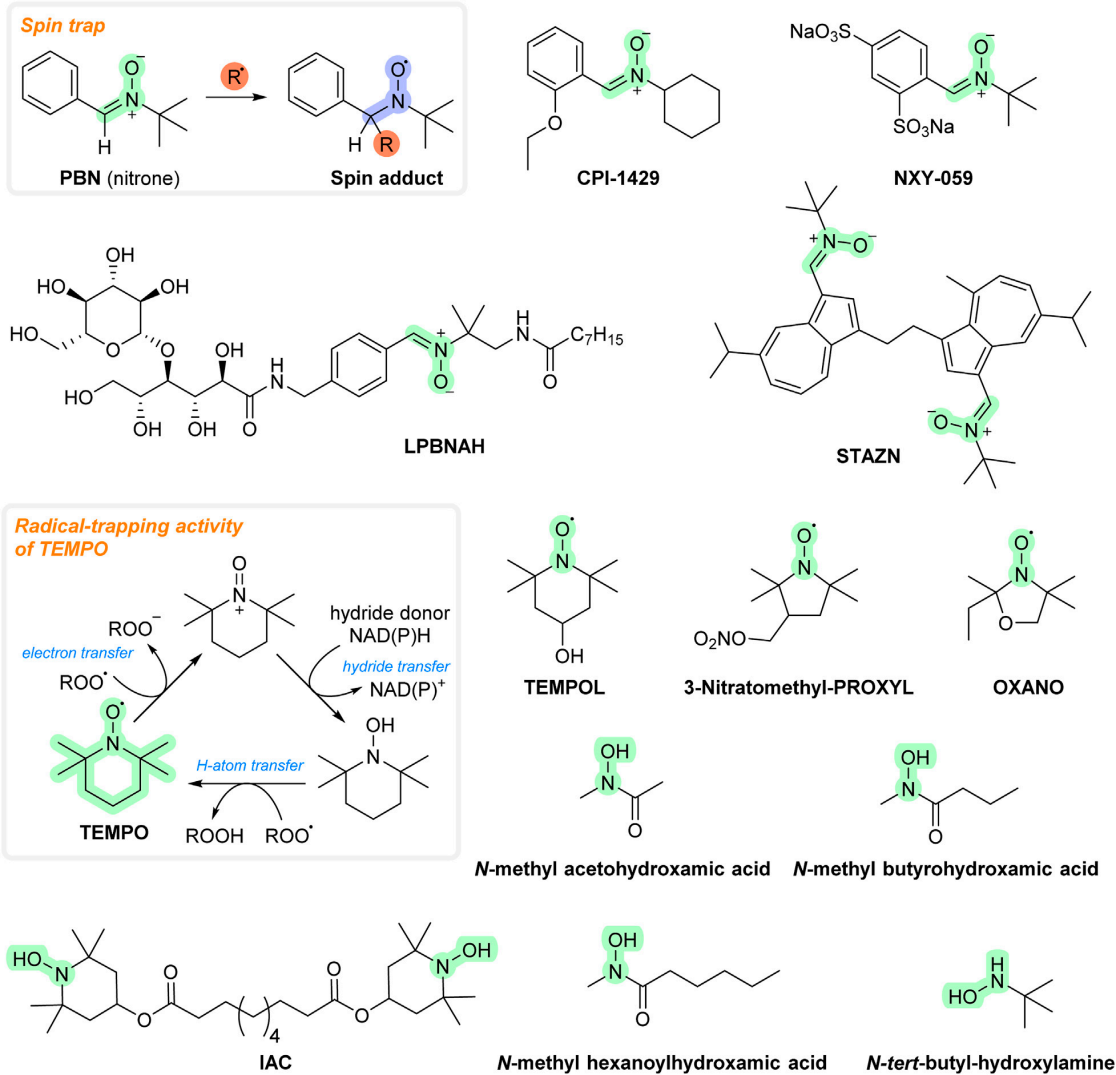
Chemical structures of spin traps/nitroxides.

The reaction products of nitroxides, hydroxylamines, may have an antioxidant effect as well. PBN undergoes degradation to benzaldehyde and *N*-*tert*-butyl-hydroxylamine, which may be a stronger AT than PBN (Atamna et al., 2000). *N*-*tert*-butyl-hydroxylamine was found to cross the BBB and showed a protective effect in a mouse model of infantile neuronal ceroid lipofuscinosis (Sarkar et al., 2013). IAC, the reaction product of TEMPO and presumably also TEMPOL, showed protective effects in animal models of various diseases, including colitis, Alzheimer’s disease, and diabetes (Novelli et al., 2007; Vasina et al., 2009; Puoliväli et al., 2011). It can cross the BBB as well (Canistro et al., 2010). Some hydroxamates, including *N*-methyl acetohydroxamic, *N*-methyl butyrohydroxamic, and *N*-methyl hexanoylhydroxamic acids, were also considered ATs and tested with several reperfusion models (Halliwell and Gutteridge, 2015). All these compounds are shown in Figure 4.

While spin traps and nitroxides may possess significant therapeutic potential, their clinical applications may be limited by poor bioavailability and targeting and inadequate BBB penetration (mainly due to low lipophilicity). To overcome these problems, various halogenated derivatives (e.g., chlorinated or boronated PBN), alkylated derivatives having a lipophilic tail (e.g., those with dodecyl chains), bifunctional derivatives (e.g., GS-PBN, Mito-DEPMPO, 5-ChEPMPO, DECPO, and 4-HMDEPMPO), conjugates with drugs or targeting molecules (such as PEG or heparin, that both can cross the BBB), dimers, and cyclodextrin polymers have been developed in the last few years (Han et al., 2009; Kleschyov et al., 2012; Wang et al., 2021; Marco-Contelles, 2024). Several nanoparticles with enhanced delivery abilities have been developed as well, which includes those attached to the silica core protected by poly(ethylene glycol) chains (PluS–NO), CdSe quantum dots, rotaxane-branched radical dendrimers (Gn-TEMPO), G4-polyamidoamine dendrimers, 3Gc0T zero-generation dendrimer, polyurethane dendrimers, gold nanoparticles, liquid crystal nanoparticles, nanosized sterically stabilized liposomes, poly[oligo(ethylene glycol)methyl ether acrylate] and poly(2-hydroxyethyl acrylate) conjugates, and redox nanoparticles. These derivatives showed better ROS scavenging ability (toward various radicals, such as H_2_O_2_) *in vitro*. Activities of these are thoroughly summarized elsewhere (Sadowska-Bartosz and Bartosz, 2024).

## 5 Glutathione peroxidase mimetics

**FIGURE 5:**
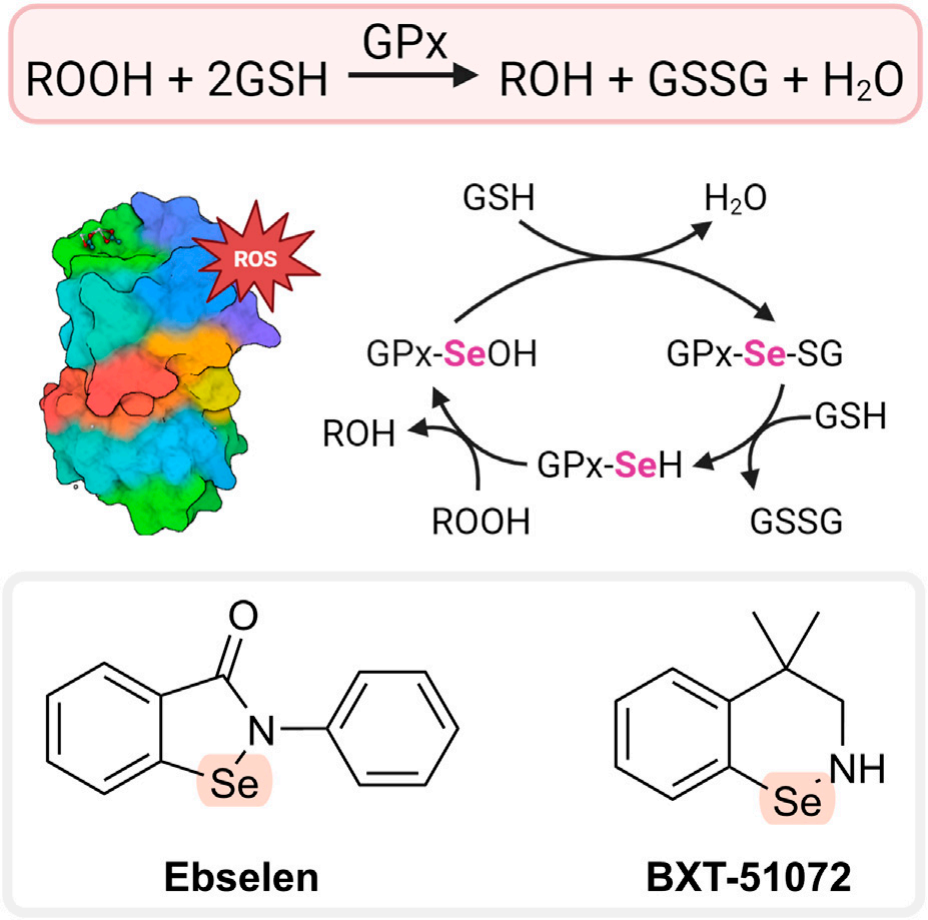
AT cycle of GPx and glutathione peroxidase mimetics. Created with Biorender.com.

The main function of the enzyme glutathione peroxidase (GPx) is the elimination of hydrogen peroxide (see Figure 5; Table 3). Selenocysteine has an important role at the active sites of GPx (Orian et al., 2015). Consequently, low-molecular-weight selenium-containing compounds that mimic GPx’s hydrogen peroxide-scavenging activity may hold therapeutic potential. Ebselen (2-phenylbenzo[*d*][1,2]selenazol-3(2*H*)-one; Figure 5) was one of the first such compounds developed (Parnham and Sies, 2013). It can decompose peroxides. Prior to this action, ebselen needs to be reduced, i.e., the Se-containing ring is opened, and the Se is converted to selenol (-SeH). The reduced selenol then reacts with peroxide, and ebselen is regenerated. Selenol can also react with another ebselen molecule to form a diselenide, which may contribute to the catalytic cycle. Some studies, however, suggested that ebselen is an inefficient catalyst due to formation of various unreactive intermediates that prevent the regeneration of the original compound (Sarma and Mugesh, 2005; Kumar et al., 2014). The main reducing agent of ebselen *in vivo* is GSH. However, other compounds may have a similar role including *N*-acetylcysteine (NAC), reduced thioredoxin, and dihydrolipoate (Parnham and Sies, 2013). Ebselen has shown positive effects with numerous animal models of diseases, including metabolic syndrome, noise-induced hearing loss, 1-methyl-4-phenyl-1,2,3,6-tetrahydropyridine (MPTP)-induced Parkinson’s disease, alcohol-induced liver injury, atherosclerosis, and diabetes (Day, 2008; Halliwell and Gutteridge, 2015). It also showed some therapeutic benefit in clinical studies of various diseases, mainly stroke, hearing loss, Meniere’s disease, and severe acute respiratory syndrome coronavirus 2 (SARS-CoV-2) (Forman and Zhang, 2021; Ramli et al., 2022; Sahoo et al., 2023); however, it was ineffective in diabetes (Beckman et al., 2016). These results indicate that ebselen (and GPx mimetics in general) could be particularly useful in neurodegenerative and respiratory disorders. Ebselen has AT properties *in vitro* against various radical species (including HOCl, singlet oxygen, and peroxynitrite). However, it also showed anti-inflammatory actions, such as inhibition of 5- and 15-lipoxygenases (LOX), NOS, and phagocyte-related ROS production (Day, 2008) (see Section 11.2). Its therapeutic activity may not involve an AT mechanism.

Ebselen has also been tested in combination with other ATs that may improve its efficacy. Together with vitamin E, it modulated the activity of acetylcholinesterase and reduced demyelinating events in different areas of rat brains (Mazzanti et al., 2009). The combined oral formulation of ebselen/allopurinol reduced multiple cisplatin toxicities in rat breast and ovarian cancers (Lynch et al., 2005). Inhalable microparticles combining remdesivir and ebselen demonstrated antiviral properties against SARS-CoV-2 infection (Saha et al., 2023). In addition, ebselen was also tested together with various antibacterials such as daptomycin, retapamulin, fusidic acid, and mupirocin and exhibited synergistic effects with these drugs (Thakare et al., 2020). Similarly, ebselen also showed synergistic effects with silver, making it more selective for pathogenic bacteria than for mammalian cells (Zou et al., 2017). There are also reports of ebselen conjugates with commonly used drugs, such as clioquinol, a compound known for its ability to chelate metal ions and inhibit Aβ deposition. The conjugate was able to penetrate the CNS without inducing toxicity *in vivo* and demonstrated inhibition of Aβ aggregation and H_2_O_2_ scavenging activity in *in vitro* conditions (Wang et al., 2016). Ebselen has also been formulated to nanoemuslsions that showed antifungal activity against vulvovaginal candidiasis in a mouse model (Menon et al., 2021). Again, the above studies suggest that ebselen may be valuable in the treatment of neurological disorders and as an antimicrobial agent in various conditions (especially respiratory disorders).

Another compound that has GPx-like activity is BXT-51072 (Figure 5). It has a better protective effect than ebselen, being several-fold more reactive in catalyzing the peroxide reaction. BXT-51072 may help with several diseases including IBD, asthma, chronic obstructive pulmonary disease, and stroke. It showed promising results in clinical trials of ulcerative colitis. Currently, its further development is not happening (May, 2016; Forman and Zhang, 2021).

Tellurium has similar chemical properties as Se. Various Te derivatives have been developed and may eliminate various radical species, such as peroxides and peroxynitrite, *in vitro* (Pariagh et al., 2005; Lu et al., 2017). Research on these compounds is still limited.

## 6 Synthetic analogs of vitamins E and C

Vitamin E (tocopherols) deficiency is associated with neurological and reproductive abnormalities. These conditions usually disappear after adequate supplementation with vitamin E (Dewick, 2009). Similarly, administration of vitamin E to infants and adults suffering from inborn glutathione deficiency have provided therapeutic benefits (although quite limited) (Halliwell and Gutteridge, 2015). However, with other diseases putatively associated with oxidative stress, including diabetes, cardiovascular disorders, cancer, and various neurodegenerative disorders, vitamin E does not appear to have a beneficial effect (Robinson et al., 2006; Steinhubl, 2008; Halliwell, 2024). Additionally, vitamin E supplementation is not entirely safe as higher doses have been associated with specific side effects, including hemorrhagic stroke (Sesso et al., 2008) and increased risk of prostatic cancer (Klein et al., 2011) (Table 1). There are several possible reasons why vitamin E is ineffective in the treatment of human diseases. Its bioavailability is very less, and it takes a relatively long time for significant concentrations of vitamin E to accumulate in the target tissues (Halliwell and Gutteridge, 2015; Halliwell, 2024). Vitamin E and its derivatives (e.g., α-tocopheryl acetate) are commonly used in the food industry as preservatives to prevent rancidity by reducing the rate of lipid peroxidation (Dewick, 2009). However, vitamin E appears to be ineffective at inhibiting lipid peroxidation in humans, or at least its efficacy may vary depending on the specific disease. Additionally, vitamin E may not be biologically important as an AT, but rather as a signaling molecule and regulator of specific gene expressions (Azzi and Zingg, 2005).

**FIGURE 6:**
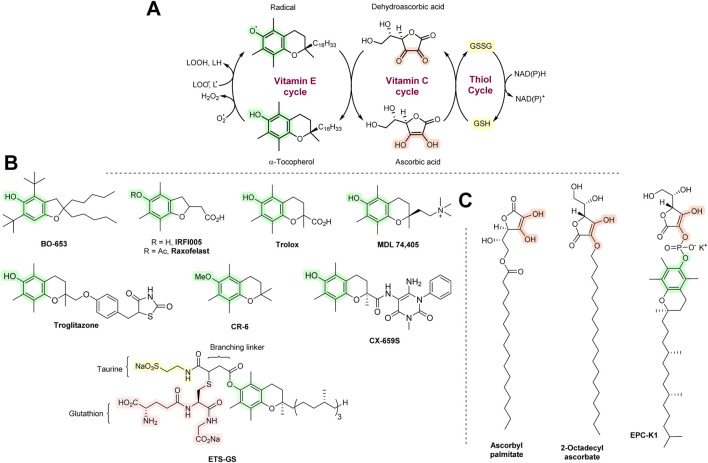
**(A)** The AT network of vitamins E and C and thiols. **(B)** Synthetic vitamin E analogs. **(C)** Synthetic vitamin C analogs.

Various structurally related derivatives of vitamin E have been developed, some of which have improved antioxidant effects with *in vitro* AT assays or animal models. Some have a benzofuran ring instead of the chroman ring of vitamin E, e.g., BO-653. It showed an antiatherosclerosis effect with animal models and lowered F_2_-isoprostane levels (that are often used as biomarkers for oxidative stress) in vitamin E-deficient mice (Halliwell and Gutteridge, 2015; Valgimigli and Amorati, 2019). BO-653 was able to suppress hepatitis C replication (Yasui et al., 2013). However, no further clinical development has taken place since then. Raxofelast is a water-soluble form of vitamin E that improves vascular endothelial dysfunction in diabetes (Hadi and Al Suwaidi, 2007) and aids wound healing (Bitto et al., 2007). It undergoes deacetylation hydrolysis to form IRFI-005 *in vivo*. IRFI-005 may have greater antioxidant action than raxofelast (Barzegar et al., 2011). Trolox is another hydrophilic analog of vitamin E that is a good scavenger of peroxyl and alkoxyl radicals. Upon reaction with them, a resonance-stabilized Trolox radical will be formed, which is presumably relatively easily regenerated by vitamin C (or natural vitamin E; Figure 6A). However, due to its high hydrophilicity, Trolox enters cells with difficulty. It is extensively used as a reference compound in a number of *in vitro* AT assays (MacDonald-Wicks et al., 2006).

MDL 74,405 contains a quaternary ammonium group. It is highly cardioselective as it is predominantly deposited in the heart relative to other tissues and blood (Chan et al., 1994; Kuo et al., 1995). It showed positive effects in dog models of heart ischemia/reperfusion (Tang et al., 1995a; 1995b). Further work has not been carried out. Troglitazone is an antidiabetic drug with AT properties, which exerts its blood sugar-lowering activity by enhancing insulin sensitivity. It shows significant hepatotoxicity as it readily oxidizes to form various radicals, including quinones (Kassahun et al., 2001). It exerted a protective effect with brain ischemia in rats. However, further work has not been carried out. CX-659S showed anti-inflammatory activity in animals (contact hypersensitivity and allergic reactions) (Inoue et al., 2003), but this has not been followed-up. ETS-GS is a combination of vitamin E, taurine, and GSH that showed protective effects with various models of ischemia/reperfusion and inflammation (Sugita et al., 2013). All agents discussed in this section are shown in Figure 6B.

Many animals can synthesize vitamin C. Some, however, including certain birds, guinea pigs, bats, primates, and humans, lack the necessary biosynthetic apparatus and must acquire vitamin C through external sources, i.e., diet. Vitamin C deficiency leads to serious health complications, including scurvy, skin lesions, fragile blood vessels, bleeding gums, and tooth loss. The beneficial effects of vitamin C are a result of its antioxidant activity, especially as it provides a regenerating system for vitamin E. However, vitamin C has other important biological roles. It is an important cofactor in the hydroxylation of proline to 4-hydroxyproline and lysine to 5-hydroxylysine, which accounts for ∼25% of the collagen structure composition. Vitamin C is also associated with hydroxylation of tyrosine in the synthesis of catecholamines (dopamine, noradrenaline, and adrenaline) and homogentisic acid, the precursor of tocopherols and plastoquinones (Dewick, 2009). Its therapeutic benefit may, therefore, not always be necessarily associated with its antioxidant action (Table 1).

Various lipophilic esters of vitamin C have been synthesized. Examples are ascorbyl palmitate and 2-octadecylascorbate. Both have been used in the food industry as food preservatives. They have also been tested as ATs with various animal disease models (Halliwell and Gutteridge, 2015). However, it seems that they are not of interest as medicinal agents. EPC-K1 is a phosphate ester derivative of vitamins C and E. It was extensively investigated with animal models of ischemia/reperfusion and stroke (Zhang et al., 2001; Kato et al., 2003; Yamamoto et al., 2011), although there have been no human clinical trials. Synthetic vitamin C analogs are shown in Figure 6C.

As indicated above, vitamins E and C and their respective derivatives have largely failed to provide therapeutic benefits in many studies because of some possible reasons. Inefficiency may be caused by limited tissue retention. For example, it is known that α-tocopherol can enter the brain, but it is unclear how far its supplementation elevates human brain levels (Halliwell, 2024). In contrast, vitamin C supplementation does not appear to increase levels in the brain at all (Terpstra et al., 2011). Even if the compound enters the target tissue/organ, it may not reach the active sites of oxidative damage (e.g., mitochondria). It may reach the correct site, but may not be targeted toward the specific agent responsible for oxidative damage, as observed in the case of vitamin E that inhibits lipid peroxidation, but may not prevent the increased oxidative DNA, RNA, and protein damage seen in Alzheimer’s disease (Praticò, 2008). Intervention with vitamin E/C derivatives could have been initiated at advanced stages of the disease and not during the onset and early stages (in other words, it was too late to be effective). High doses of vitamin E/C derivatives were studied. Some *in vitro* studies suggest that high doses of vitamin E and C can be pro-oxidant in nature (Witting et al., 1999; Pearson et al., 2021), and this may also apply for the semisynthetic variants. Studies may also have used high doses of one agent, which may have resulted in a reduced uptake and/or distribution of other substances with important metabolic activity. For example, high doses of a semi-synthetic variant of vitamin E/C could have lead to reduced levels of endogenous natural tocopherols. There may be also issues with instability and degradation (vitamins E and C are quite prone to degradation in solutions) or low interaction of the semi-synthetic derivatives with the delivery systems (such as sodium-dependent vitamin C transporters).

To overcome some of these limitations, several advanced formulation strategies have been developed to increase the bioavailability, stability, and targeted delivery of these vitamins and their semisynthetic forms. These include the incorporation of the vitamins to the liposomes (Marsanasco et al., 2011), poly(lactic-co-glycolic) acid (PLGA) nanoparticles (Astete et al., 2011), solid lipid nanoparticles (Gambaro et al., 2022), nanostructured lipid carriers (Saez et al., 1984), chitosan carriers (Sherif et al., 2024), nanocapsules (Khayata et al., 2012), cyclodextrin complexes (Celebioglu and Uyar, 2017; Khan et al., 2023), and peptides and antibody conjugates (Jung et al., 2018). These systems may represent new horizons for compounds that have not been clinically tested thus far or that have previously failed in clinical settings as potential ATs. Whether these advanced formulations solve the above discussed issues of vitamin E and C analogs remains to be known.

### 6.1 Other chain-breaking ATs

Probucol was primarily designed and approved for medicinal use as a cholesterol-lowering agent in the treatment of coronary artery disease, and secondarily, it may provide antioxidant activity. In animal disease models, probucol protected the heart from doxorubicin-induced toxicity (El-Demerdash et al., 2003). Probucol decreases high-density lipoprotein (HDL) and has been withdrawn from therapeutic use. Succinobucol (AGI-1067) is a succinate ester derivative of probucol showing decreased HDL-lowering ability. Like probucol, succinobucol had antioxidant activity. It had advanced into clinical trials as an anti-atherosclerotic agent, although it failed in a phase III trial (Muldrew and Franks, 2009).

Coenzyme Q reportedly has positive effects in neurodegenerative diseases (e.g., Alzheimer’s and Parkinson disease). The therapeutic benefit may be related to its AT mechanism, although there is still little direct evidence. Some of the coenzyme Q derivatives, such as idebenone or vatiquinone (EPI-743), have been tested with various models and even subjected to human clinical trials of neurodegenerative diseases, and they seem to have only limited benefit (Gutzmann et al., 2002; Zesiewicz et al., 2018). Idebenone is orally active and has demonstrated some side effects that include vomiting, stomach pain, loose stools, fast heart rate, or increased risk of infection. OPC-14117 has shown promising results in animal models of neurodegenerative disorders (Abe et al., 1997). However, it did not have therapeutic benefits in patients with Huntington disease (Dickey and La Spada, 2018). BN-82451 showed some benefit in Huntington’s (Klivenyi et al., 2003) and Parkinson’s disease (Spinnewyn et al., 2011) and amyotrophic lateral sclerosis (Chabrier and Auguet, 2007) patients. It has not been tested in humans. Again, its mode of neuroprotection may not be related to its AT mechanism as it also demonstrates Na^+^ channel blockage and COX-inhibitory activities. Various structurally related analogs to BN-82451 include LY-178002, LY-256548, ONO-144, and MK-477. Both LY-178002 and LY-256548 inhibited lipid peroxidation. They are orally active and have been tested in animal models of rheumatoid arthritis and cerebral ischemia/reperfusion. ONO-3411 and MK-477 have shown anti-inflammatory activity in mouse models, which appears to be exerted through COX inhibition. In addition, ONO-3411 showed some degree of benefit in animal models of cardiovascular and cerebral ischemia/reperfusion (Halliwell and Gutteridge, 2015). Since research on these compounds is limited, their toxicity is also largely unknown.

Phenothiazine derivatives have strong biological activities and have a long history of use as medicinal agents. Promethazine and chlorpromazine administration caused major changes in the treatment of allergy and psychiatric disorders, respectively. Promethazine acts as an antagonist to the H_1_ receptors for histamine. Apart from allergies, it is also used to treat insomnia, nausea, and for sedating agitated or anxious patients. Promethazine was also shown to inhibit lipid peroxidation (Poli et al., 1989). Chlorpromazine is a dopamine receptor antagonist and is used to treat schizophrenia, bipolar disorder, attention-deficit hyperactivity disorder (ADHD), anxiety, nausea, and vomiting. It undergoes a conversion *in vivo* to hydroxylated products that may exert antioxidant activity (Eluashvili et al., 1978). Both promethazine and chlorpromazine have major side effects, such as drowsiness, headaches, nightmares, dizziness, light-headedness, restlessness, confusion, irregular heartbeat, and fainting.

*N,N′*-Diphenyl-*p*-phenylene diamine (DPPD) is used as an AT in the lubricant and polymer industries. It is sometimes used as an inhibitor of lipid peroxidation in animal studies (Tangirala et al., 1995). Ethoxyquin is a quinoline-based antioxidant developed by Monsanto in the 1950s, primarily used as a food preservative (E_324_) to prevent browning in fruits, especially pears. However, use of ethoxyquin as a food additive has been banned in many countries due to evidence of various toxic effects in animal studies, including mutagenic activity. Despite these restrictions, it is still sometimes used in animal feeds, such as pet food and fish meals, which may result in ongoing human exposure. Ethoxyquin is also experimentally tested in longevity studies (Błaszczyk et al., 2013; Aquilina et al., 2015; Shaw, 2018). 5,8-Dimethyl-9*H*-carbazol-3-ol (HDC) exhibits inhibition of lipid peroxidation. Its structurally related compound, carvedilol, is an antihypertensive medication that also demonstrates lipid peroxidation inhibitory activity *in vitro* (Halliwell and Gutteridge, 2015). Furthermore, 3-hydroxycarvedilol (SB-211475) shows even stronger inhibitory effects. Its administration decreased levels of 4-hydroxynonenal (a product of lipid peroxidation) in patients with cardiomyopathy (Malig et al., 2016). However, it failed to affect urinary levels of F_2_-isoprostanes in healthy volunteers (Fahlbusch et al., 2004). SUN-N8075 is claimed to block sodium and calcium channel activity (as its precursor flunarizine) as well as to have an antioxidant effect. It has protective effects in light-induced retinal damage in rats (Ojino et al., 2014).

An isomeric mixture of 2-*tert*-butyl-4-methoxyphenol and 3-*tert*-butyl-4-hydroxyanisole (BHA), butylhydroxytoluene (BHT), and *tert*-butylhydroquinone (TBHQ) are chain-breaking ATs extensively used in the food industry as preservatives. All are considered endocrine disruptors and carcinogens (Yang et al., 2018). They show toxicity at high concentrations, beyond what is used in food. In addition, carcinogenic properties have been demonstrated in animal models that are not fully relevant to humans (Halliwell and Gutteridge, 2015; Shaw, 2018). Although these compounds are strong antioxidants and also exhibit other activities, such as antimicrobial effects, research into their therapeutic potential has been limited. However, recent studies suggest they may help suppress various types of inflammation. (Delanghe et al., 2021; Liu et al., 2023). Various analogs of these compounds have been developed with improved AT properties, such as BHT attached to carbon nanotubes (Lucente-Schultz et al., 2009). Propofol is used to initiate and maintain general anesthesia and sedation in patients undergoing surgery. It is also used in cases of epilepsy where conventional agents failed to provide the expected benefit. Since propofol structurally resembles BHT, it may also exhibit antioxidant effects at concentrations comparable to those used during anesthesia. However, the relevancy of its antioxidant activity *in vivo* is still questionable and warrants further research (Murphy et al., 1992; Braz et al., 2015; Han et al., 2022).

Propyl gallate (EU code E_310_) is an ester formed by the condensation of gallic acid and propanol. It has been used since 1948 as an additive for foods containing oil and fat to slow down lipid peroxidation. In addition, it inhibits lipoxygenase activity (Georgieva et al., 2013) and was shown to bind iron ions and to reduce Fe^3+^ to Fe^2+^ (Binbuga et al., 2005). There are different opinions in the literature about the toxicity of propyl gallate. Some studies have suggested that propyl gallate may cause cancer (in several organs) in rats and act as an endocrine disrupter (in a similar manner to BHT, BHQ, and TBHQ) (Ham et al., 2019; Esazadeh et al., 2024), while other authors claim that it is generally safe (Shaw, 2018).

Many synthetic additives in the food industry are regarded as having toxic properties, and there is still a tendency to replace them with natural “non-toxic” agents such as flavonoids. These compounds are also therapeutic agents in the treatment of oxidative stress-related diseases. However, flavonoids are not used for development of pharmaceutical drugs, as reviewed elsewhere (Halliwell, 2012; Tauchen et al., 2020; Cheah and Halliwell, 2021). However, there may be a few exceptions. Daflon is a micronized fraction consisting of diosmin (90%) and hesperidin (10%) that is used to treat chronic venous insufficiency. Its clinical efficiency is still dubious. The mechanism of action remains to be elucidated and may not be related to antioxidant activity (Lyseng-Williamson and Perry, 2003). Silybin is used for cases of liver disease and injury. It, however, remains peripheral to mainstream medicine. Silybin bis-hemisuccinate injection is of value in the treatment of death cap (*Amanita phalloides*) poisoning (Tauchen et al., 2020). Silybin and other related flavonolignans may act as ATs, although there is now evidence that they also act on the cellular membrane of hepatocytes, inhibiting the absorption of toxins (Dewick, 2009). Other silybin derivatives are in development, such as the silybin–phosphatidylcholine complex, which has shown some efficacy with treatment of non-fatty liver disease (Ferenci, 2016).

**FIGURE 7:**
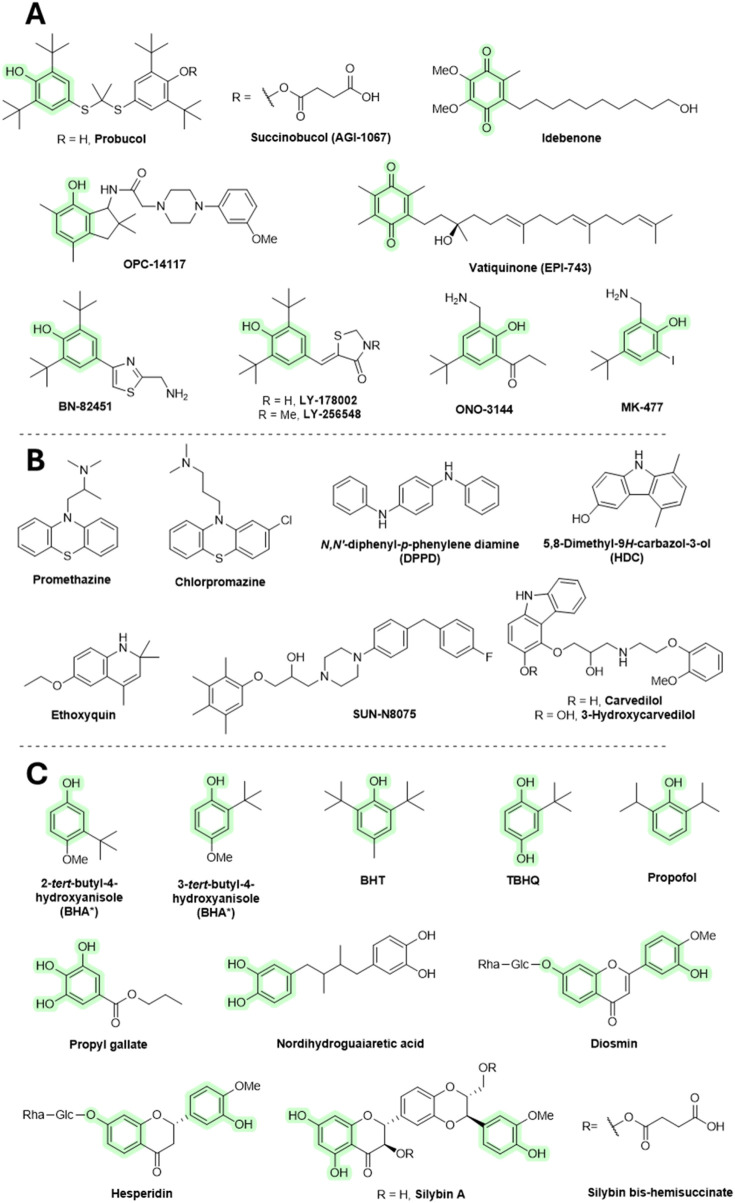
Chain breaking AT. (^*^BHA consists of a mixture of two isomers.). **(A,B)** ATs tested in a medicinal context. **(C)** ATs of value in the food industry.

Nordihydroguaiaretic acid is a lignan found in the creosote bush (*Larrea tridentata*; Zygophyllaceae). It shows AT and anti-inflammatory activity *in vitro* and in animal models. Its activity is related to 5-LOX inhibition (Gilbert et al., 2020). It also binds iron and reduces Fe^3+^ to Fe^2+^ (Manda et al., 2020). Nordihydroguaiaretic acid has been (since the 1950s) as a food preservative. However, it was withdrawn in the 1960s due to reported renal and hepatic toxicities. It is still available in some countries as a dietary supplement (Arteaga et al., 2005). All compounds discussed in this section are shown in Figure 7.

Chain-breaking ATs are a relatively interesting group of compounds that perhaps contain the most number of examples that are valuable in both the food and medical industries. Given that many chain-breaking antioxidants used in the food industry structurally resemble those used in medicine, it has been proposed that these food antioxidants might serve a dual purpose: acting both as preservatives and as antioxidants beneficial to consumers. However, while their antioxidant effects have been extensively tested in terms of food stability, studies investigating their impact on human health remain limited. As indicated above, many of these substances also show toxic effects, for which they have been withdrawn from use or withdrawal is being considered. While chain-breaking ATs are associated with short-term use of higher doses in the medicinal industry, in the food industry, it is exactly the opposite (long-term use of trace amounts). From this perspective, food ATs are generally considered safer. However, it is also questionable whether they can provide therapeutic benefit *via* antioxidant effect at these low concentrations. On top of that, many food chain-breaking ATs will have lower rates of reaction with ROS and limited recycling ability (e.g., BHT and flavonoids). Even in the case of propofol, which is used in relatively large doses, the achievement of antioxidant activity may be relatively problematic (Han et al., 2022). Additionally, the general antioxidant mechanism of chain-breaking antioxidants involves the inhibition of lipid peroxidation. However, this effect observed in food preservation may not fully translate to *in vivo* conditions as some food antioxidants have demonstrated efficacy in preventing lipid peroxidation in foods, but not necessarily in human disease contexts. (e.g., vitamin E) (Dewick, 2009). Except for propofol, all the compounds listed in this section are orally active, which could facilitate their potential wider use. Furthermore, the problems associated with vitamin C and E derivatives (e.g., limited tissue retention, targeting to the active site of oxidative stress, no targeting to specific ROS, and poor interaction with specific carriers) will largely also apply to these substances (especially because they structurally resemble vitamin C and E and share similar mechanisms of action). Similarly, to enhance the efficacy of chain-breaking antioxidants, new derivatives with reduced toxicity and improved effectiveness, such as novel synthetic variants, various conjugates, or nanoformulations, specifically targeted to active sites of oxidative stress and particular ROS need to be developed. However, reports on these advanced formulations on chain-breaking ATs discussed in this section are still very limited.

## 7 Lazaroids

Various drug classes have been suggested as candidates to develop analogs of commonly used pharmaceuticals with enhanced AT properties, including hypolipidemic agents, anesthetics, non-steroidal anti-inflammatory drugs, or antiarrhythmic agents. Methylprednisolone exhibits anti-inflammatory activity by inhibiting the phospholipase enzymatic activity. It also inhibited lipid peroxidation in the brain during traumatic events. These results led to the development of lazaroids (21-aminosteroids), in which the steroid skeleton has been improved with addition of an AT component (Kavanagh and Kam, 2001). Many lazaroid derivatives have been developed that inhibit iron-dependent lipid peroxidation in brain cells *in vitro*, as well as show neuroprotective activity in various animal models of traumatic brain injury (TBI) and of the spinal cord. Perhaps the most studied lazaroid is the tirilazad mesylate (Freedox; U-74006F, U series named after the Upjohn Company). It was subjected to several clinical trials for stroke (Putman et al., 1994), spinal cord injury (Bracken et al., 1998), and TBI (Marshall and Marshall, 1995). However, it failed to provide any notable therapeutic benefit in most patients and in some even produced adverse effects. However, tirilazad mesylate may be helpful in treating subarachnoid hemorrhage in men, but not in women, as it appears that women metabolize it much faster (Cahill and Hall, 2017). There is little evidence that lazaroids function as ATs *in vivo*; therefore, their therapeutic benefits are likely due to other mechanisms.

**FIGURE 8:**
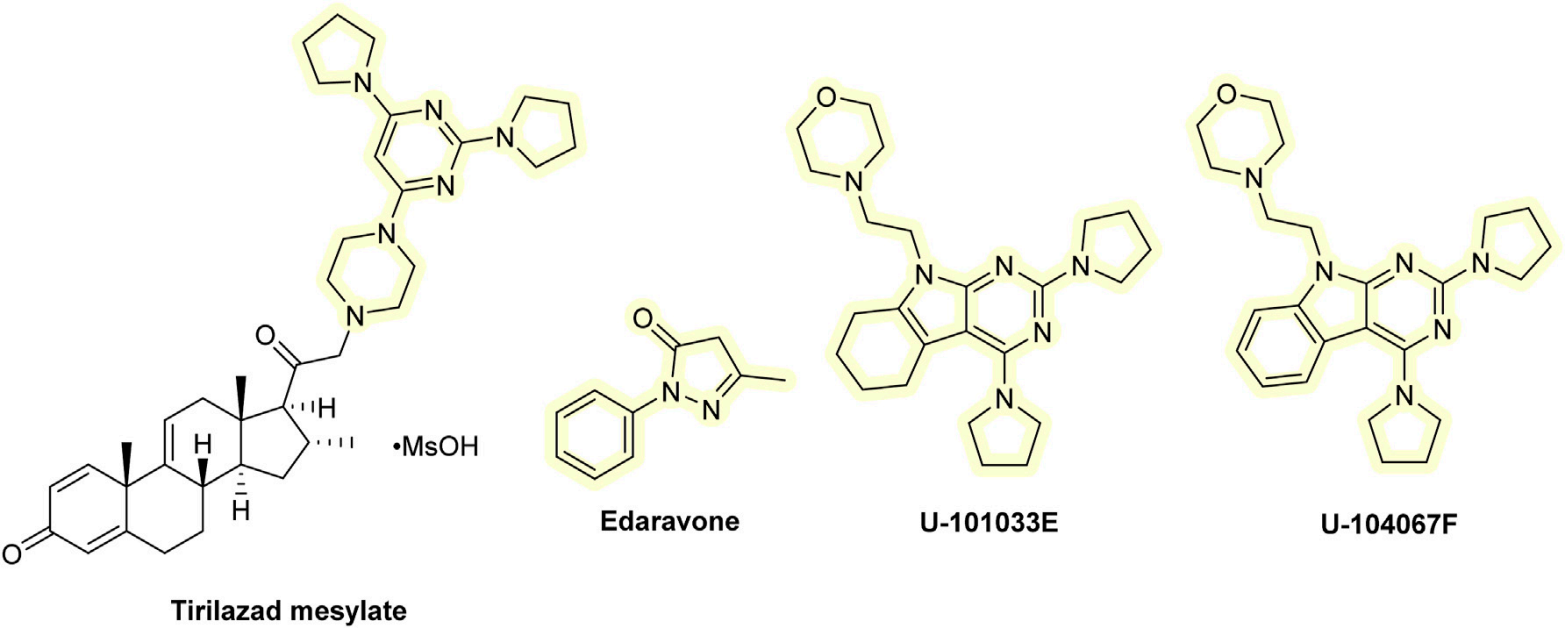
Chemical structures of lazaroids.

Tirilazad mesylate accumulates at the BBB and has limited penetration into the brain . Some related derivatives, such as U-101033E or U-104067F, cross the BBB more readily (Halliwell and Gutteridge, 2015). So far, they have not been tested in clinical studies. However, preclinical data on animals (gerbils) showed that both U-101033E and U-104067F can significantly attenuate the post-ischemic loss of dopaminergic nigrostriatal neurons (Andrus et al., 1997). Another analog that, like tirilazad mesylate, has been intensively tested in neurological conditions is edaravone. It has shown some therapeutic benefits in clinical trials of stroke and amyotrophic lateral sclerosis (Miyaji et al., 2015; Kobayashi et al., 2019; Soares et al., 2023). In some countries, it is used to treat amyotrophic lateral sclerosis and is administered to patients after stroke to facilitate their recovery. Edaravone is the only member of the lazaroid compound which is used clinically as an AT (Soares et al., 2023; Petrov et al., 2017). It may also act as an anti-inflammatory drug owing to its beneficial effects in animals with inflammatory conditions (Yuan et al., 2014). Additionally, the available clinical trials did not measure the biomarkers of oxidative damage, so its mechanism of action may not be indeed related to the antioxidant effects. The clinical efficacy of edaravone has been thoroughly reviewed elsewhere (Halliwell and Gutteridge, 2015; Halliwell, 2024). Even though edavarone seems to be of value in neurological disorders, it has limited ability to cross the BBB. Quite recently, some edaravone analogs with improved BBB penetration and overall efficacy were developed. For example, intranasal administration of edaravone PLGA nanoparticles showed improved brain stability and bioavailability and reduced H_2_O_2_-induced oxidative stress toxicity in mouse microglial cell line BV-2 (Lu et al., 2023). Edaravone-MIL-53(Cr) nanoparticles alleviated brain injury and cognitive dysfunction in mice receiving whole-brain irradiation (Li et al., 2025). It was also conjugated with several drugs, including glutathione, in the form of poly(methacrylic) nanogel, which had improved targeted delivery to the brain and elevated cognitive function in Wistar rats (Mozafari et al., 2023). Administration of an ionic liquid formulation of edaravone, combined with tetrabutylphosphonium cation, resulted in prolonged blood circulation time and reduced kidney distribution. It also demonstrated cerebroprotective effects comparable to those of edaravone in a rat model of cerebral ischemia/reperfusion injury (Fukuta et al., 2023). A series of edaravone derivatives bearing *N*-benzyl pyridinium moieties demonstrated significant *in vitro* acetylcholine inhibitory activity (with low activity toward butyrylcholinesterase) and antioxidant effect (Zondagh et al., 2020). Edaravone was also used in combination with the anti-inflammatory drug dexborneol and showed promising results in clinical trials of stroke. This combination was even more effective than edaravone alone (Xu et al., 2021). Structures of lazaroids are shown in Figure 8.

## 8 Thiols

Glutathione (GSH) is an endogenous AT that is clinically useful. It provides the substrate for glutathione peroxidase (GPx; Figure 4 in Section 5) but can also directly scavenge several ROS, including peroxynitrite, HOCl, and OH^•^ (Tambyraja et al., 2007). It also protects tissues against the effects of various cytotoxic agents, such as cyclophosphamide (Lopez and Luderer, 2004). GSH also protects the lungs against RS-induced damage (e.g., in cystic fibrosis, where patients suffering from this condition tend to have lower GSH levels) (Bozic et al., 2020). However, despite having endogenous AT activity, GSH may induce serious side effects. For example, it caused bronchoconstriction in asthma patients (Halliwell and Gutteridge, 2015).

GSH does not readily enter cells. Some derivatives with improved membrane-crossing properties were developed, including methyl, isopropyl, and ethyl monoesters. Upon entering the cells, they are hydrolyzed to glutathione. Diethylester derivatives were seen to be more easily delivered into cells (Cacciatore et al., 2010).

**FIGURE 9:**
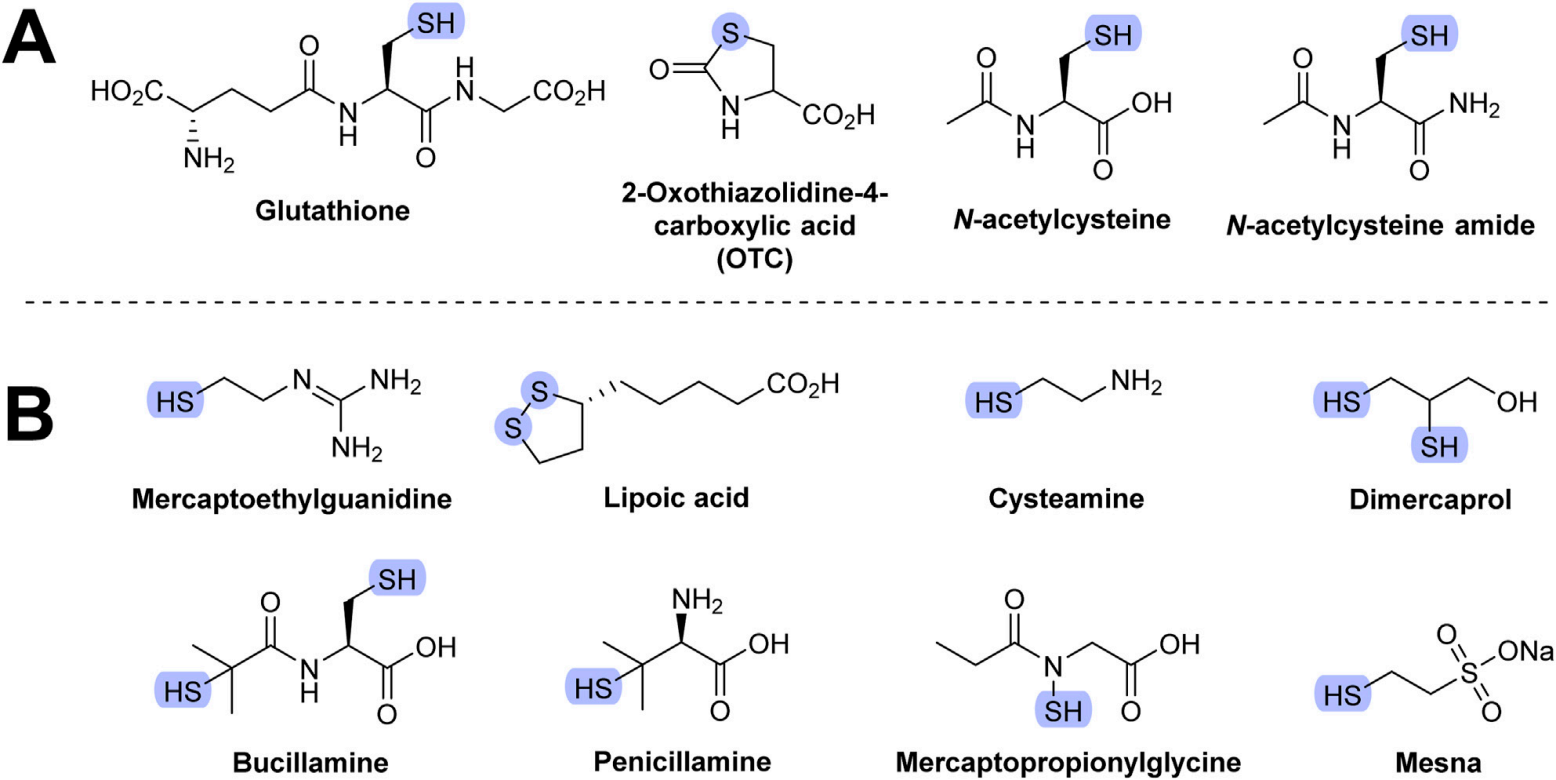
Molecular structure of thiol-based AT. **(A)** Most frequently studied thiols. **(B)** Other thiols.

2-Oxothiazolidine-4-carboxylic acid (OTC) is hydrolyzed to cysteine *in vivo*. Administration of OTC may increase endogenous GSH synthesis as cysteine is one of the three amino acids of GSH. The therapeutic efficiency of OTC is ambiguous. It decreased allergen-induced airway injury in an animal model of asthma. However, it showed no benefit in reducing oxidative stress in HIV patients (Halliwell and Gutteridge, 2015). Structures of compounds discussed in this section are shown in Figure 9A.

*N*-acetylcysteine (NAC) is used as a standard reference compound in many AT assays and is claimed to be effective in the treatment of paracetamol (acetaminophen) overdosage and toxicity. The mode of therapeutic action of NAC is believed to involve its hydrolysis in the affected cells to cysteine, leading to increased synthesis of GSH. NAC is also capable of scavenging various radicals (Forman and Zhang, 2021). However, these results were obtained using methods with serious limitations (e.g., based on boronate or dichloroflorescin diacetate probes) and may not be relevant *in vivo*, as previously reviewed elsewhere (Murphy et al., 2011; Halliwell, 2012). NAC was also shown to have non-AT activities, including interaction with receptor for *N*-methyl-D-aspartate (NMDA) and α-amino-3-hydroxy-5-methyl-4-isoxazolepropionate (AMPA) and inhibition of NF-κB activity. Its therapeutic efficiency is also dubious. It has been tested for the treatment of various respiratory disorders (e.g., bronchopulmonary dysplasia in infants) (Ahola et al., 2003). However, it showed contradictory results. NAC has also been tested for the prevention of cardiovascular disorders in patients with kidney failure, where the results were more convincing (Ye et al., 2021). However, in one study, the observed positive effect was not associated with a reduction in urinary F_2_-isoprostane levels (Efrati et al., 2003), suggesting that NAC’s protective action may involve mechanisms other than antioxidant activity. This is consistent with the fact that NAC, like most thiols, is a poor scavenger of hydrogen peroxide and superoxide (Winterbourn and Metodiewa, 1999). NAC also reduced the rate of lung deterioration in patients with idiopathic pulmonary fibrosis (Demedts et al., 2005). Few NAC derivatives have been developed, the most notable being *N*-acetylcysteine amide (AD4). It can cross the blood–brain barrier (unlike NAC). AD4 may have therapeutic benefits in various neurodegenerative disorders and inflammatory conditions (Sunitha et al., 2013).

### 8.1 Other thiols

Mercaptoethylguanidine has strong antioxidant activity against various radicals (e.g., peroxynitrite). It showed therapeutic benefits with several animal models of inflammation (Cuzzocrea et al., 1998). Lipoic acid shows antioxidant activity *in vitro,* and the intravenous form has been used to treat diabetic neuropathy in some countries (e.g., Germany). More recent clinical trials have found no difference in comparison to a placebo (Javed et al., 2015). Administration of lipoic acid to rats decreased the age-related decline of GSH levels. Various structurally related analogs of lipoic acid were developed, some of which are able to cross the blood–brain barrier (Guillonneau et al., 2003; Koufaki et al., 2007).

Several thiols have been tested for their ability to protect cells and animals against ionizing radiation, a source of oxidative stress. These include GSH, cysteine, bucillamine, cysteamine, dimercaprol, penicillamine, 2-mercaptoethanesulfonic acid (mesna), and amifostine.

Bucillamine has anti-inflammatory properties and is used in Asia as an antirheumatic drug. It is a possible medication for reperfusion injury and COVID-19 (Frank, 2022). Bucillamine is a strong thiol donor and was found to be up to 16-fold more efficient than NAC in restoring GSH levels. This is the suggested mechanism by which it prevents injury of various tissues and organs, including injuries from radiation (Horwitz, 2003).

Cysteamine is used to treat cystinosis, a condition characterized by the abnormal accumulation of cystine, the oxidized dimer of cysteine (Shams et al., 2014). The mechanisms of tissue damage in this disease are not fully understood, although it is believed that increased intracellular cystine leads to alteration of GSH levels (Levtchenko et al., 2005). Cysteamine is also used as a skin depigmenting agent in the treatment of various hyperpigmentation skin disorders (Hsu et al., 2013).

Dimercaprol (British anti-Lewisite) is used to treat heavy metal toxicity, especially that of arsenic. Dimercaprol acts by competing with toxic metals for the thiol site of the target enzymes, thus preventing the formation of metal–enzyme complexes. Toxic metals are subsequently excreted in the urine. Dimercaprol is toxic and may also chelate with toxic metals, which can result in their accumulation in certain organs (e.g., brain and testes) (Flora and Pachauri, 2010). Since dimercaprol also readily chelates copper, it is used in the treatment of Wilson’s disease, a hereditary disease in which excess copper is accumulated in the body (Leggio et al., 2005).

Penicillamine has similar medicinal indications as dimercaprol. It has replaced dimercaprol in the treatment of acute arsenic poisoning. It is primarily used to treat Wilson’s disease. Penicillamine was also shown to bind Fe^2+^ (Leggio et al., 2005). Chelating agents are discussed in Section 10.

Mesna is a chemotherapy adjuvant drug used by patients taking ifosfamide or cyclophosphamide. Mesna detoxifies urotoxic metabolites formed from these two agents (e.g., oxazaphosphorine and acrolein), and by doing so, decreases the risk of bladder bleeding. However, the combined administration of these anticancer drugs and mesna leads to increased urinary excretion of cysteine, GSH, and homocysteine (Pendyala et al., 2000).

Mercaptopropionylglycine is used in the treatment of cystinuria, a rare autoimmune condition characterized by excessive occurrence of cystine in urine, resulting in the formation of cystine stones in the urinary tract. It shares a similar chemistry and pharmacology with penicillamine. Mercaptopropionylglycine showed protective activity against cardiac reperfusion/ischemia injury (Bartekova et al., 2018).

Amifostine is hydrolyzed *in vivo* to WR-1065, which is also clinically used during radiotherapy as a protectant (WR stands for Walter Reed Army Hospital where this compound, and many other of the WR series, were developed) (Nair et al., 2001).

Although many of these agents are clinically useful, their mechanism of action may not be related to the antioxidant effect. Their structures are shown in Figure 9B.

To summarize, thiols have found their use in treatment of a plethora of human diseases; however, their use is associated with many problems. Generally, it can be said that thiols are rapidly eliminated from the blood with a short half-life. Elimination occurs *via* distribution throughout the tissues and intracellular uptake or by rapid renal excretion (Brock et al., 1984). Some thiols have poor bioavailability when taken orally and are degraded in the gastrointestinal tract (e.g., due to enzymatic degradation as in the case of glutathione and γ-glutamyltransferase) (Schmitt et al., 2015). Many thiol-based ATs may be poorly absorbed by cells, as seen again in the case of glutathione (Cacciatore et al., 2010). This might be associated with the high hydrophilicity of these substances. Many thiol-based ATs may lack tissue specificity, making it difficult to achieve therapeutic concentrations in the tissue/organ where the oxidative damage occurs (this phenomenon is also observed for other ATs discussed in this review). The toxicology of many thiols is still not fully known, and they can potentially be harmful. NAC is the most studied thiol, yet its toxicity is not entirely clear. A recent study has indicated that overdose (intraperitoneal dose of ≥800 mg/kg body weight) of NAC causes organ dysfunction, fatty liver, renal tubular necrosis, splenic damage, and even death in mice (Tsai et al., 2024). Additionally, thiols are chemically reactive and prone to oxidation, particularly in aqueous and oxygenated environments, leading to the formation of various oxidation products, including disulfides (which may be inactive), such as in the case of NAC (Primas et al., 2023; Ravi et al., 2023). Some of these by-products can also pose potential toxicity.

Like in other ATs, these negative properties can be improved/suppressed with the use of advanced techniques. These, for example, include prodrug strategies, as seen in the case of OTC, which was designed to have improved bioavailability and stability (it is thought of being converted to cysteine intracellularly, which is then used for GSH synthesis) or NAC amide that has improved lipophilicity and cell permeability. NAC ruthenium tricarbonyl conjugated prodrug is a newer discovery that has been shown to have better bioavailability. It also inhibited nitric oxide formation and the tumor necrosis factor alfa (TNF-α) expression without producing ROS itself (Seixas et al., 2015). Relative success has been observed with nanotechnology. NAC-loaded PLGA nanoparticles administered via nasal inhalation demonstrated increased deposition and better targeted delivery toward the lung (Puri et al., 2022). The GSH–cyclodextrin nanoparticle complex improved the immune response against *Mycobacterium avium* infection in human subjects (Sasaninia et al., 2023). Conjugation of cysteine and its derivatives (including NAC) to polymers (hyaluronic acid, chitosan, alginate, polyesters, polyurethanes, poly(ethylene glycol), poly(acrylic acid), polycarbophil, and carboxymethyl cellulose) significantly increased their tissue adhesion, particularly mucoadhesion, stability at physiological pH, drug encapsulation efficiency, drug release, and drug permeation. These conjugates also had non-toxic effects toward various cell lines (Chrószcz-Porębska and Gadomska-Gajadhur, 2024). Various metal–organic frameworks (MOF) have also been developed. Administration of cysteine-based MOF increased the intracellular level of cysteine and the overall antioxidant capacity in A549 lung cells (Wiśniewski et al., 2018). These strategies have only been used in few thiol-based agents. If they are also used in other thiol ATs, this can potentially lead to removing their limitations (at least some of them) and enhancing their therapeutic potential.

## 9 AT targeted against the mitochondria

**FIGURE 10:**
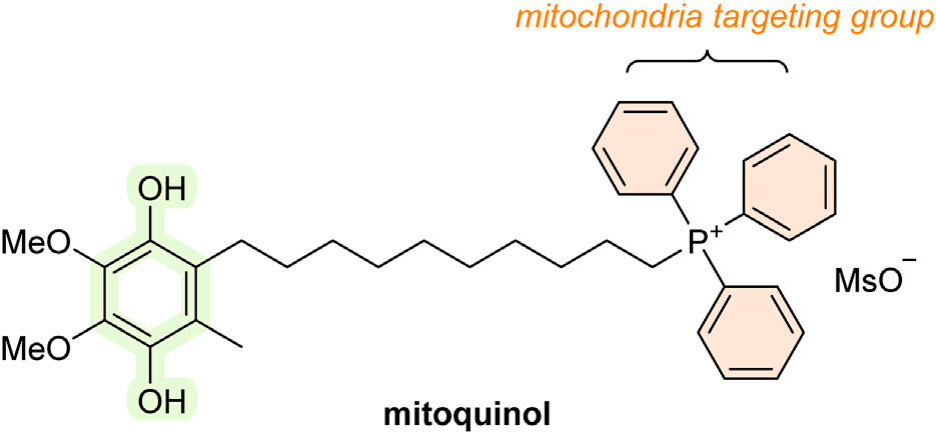
Structure of mitoquinol (MitoQ_10_; mitochondria-targeted AT).

The mitochondria are significant producers of reactive species, and mitochondrial damage is one of the major contributors to the onset of various diseases and aging. Therefore, developing antioxidants specifically designed to selectively target the mitochondria could be beneficial for treating the aforementioned conditions. Indeed, research attention in the last decade has focused significantly on antioxidants targeting the mitochondria, and it appears that the focus is shifting from more traditional antioxidants (see Figure 2). One approach in creating a mitochondria-targeted AT is to bind an antioxidant molecule (e.g., SOD, thiol, vitamin E, BHT, ebselen, CoQ, or various spin traps, such as TEMPO) with a hydrocarbon chain of varying length to the phosphorus of the lipophilic compound triphenylphosphonium (TPP) such as mitoquinol (MitoQ_10_; Figure 10). These compounds accumulate in the mitochondria and were shown to protect cell cultures from damage caused by the addition of peroxide or hypoxia. Animal studies reveal that they can enter all tissues, including the brain. MitoQ_10_ is the most studied of them all. MitoQ_10_ has been investigated in phase II clinical trials in humans for the treatment of Parkinson’s disease and chronic hepatitis C (Kezic et al., 2016). In addition, it also showed therapeutic benefits in various animal disease models, including cardiovascular disorders (Silva et al., 2016); conditions involving ischemia/reperfusion (Kezic et al., 2016); neurodegenerative disorders, e.g., Alzheimer’s (Oliver and Reddy, 2019) and Parkinson’s disease (Liu and Wang, 2014); and traumatic brain injury (Ismail et al., 2020). However, the results are not conclusive, and some studies have observed no protective effects at all (Adlam et al., 2005). Mitochondria-targeted antioxidants can be valuable tools for studying the effects of mitochondrial ROS removal on both normal physiology and pathological conditions. Compounds like TPP and similar molecules can be used to make ROS-detecting agents, such as dihydroethidium, mitochondria-specific (Smith et al., 2012; Halliwell and Gutteridge, 2015).

## 10 Metal chelators

Various chelating agents have been used to inhibit metal-dependent oxidative damage. Metals like iron and copper are considered significant contributors to oxidative stress. The antioxidant action of chelators may be achieved using different mechanisms. These include the binding of a metal to a given AT, leading to decreased production of RS or the ability to scavenge RS. The former mechanism is preferred because RS scavengers can be consumed during the reaction and may generate toxic radicals derived from the chelators (Halliwell and Gutteridge, 2015).

One of the first chelating agents documented to reduce RS production (specifically the hydroxyl radical) *in vitro* was diethylenetriaminepentaacetic acid (DTPA). The antioxidant action of DTPA is derived from its ability to chelate Fe^3+^ and thus prevent its reaction with superoxide and hydrogen peroxide, leading to formation of hydroxyl radicals (i.e., preventing Haber–Weiss and Fenton’s reactions) (Cohen and Sinet, 1982). The resulting molecule, Fe^3+^–DTPA is slowly reduced by superoxide, which results in the production of fewer hydroxyl radicals (Rahhal and Richter, 1988). However, hydroxyl radical may still be formed, i.e., during the reaction of Fe^2+^–DTPA with hydrogen peroxide (Cohen and Sinet, 1982; Egan et al., 1992). Superoxide is not the only reducing agent of the Fe^3+^–DTPA complex. It may be reduced by other, more powerful agents (Sutton and Winterbourn, 1984). DTPA, therefore, is not a general inhibitor of iron-dependent hydroxyl radical production. DTPA is currently used only marginally as it causes depletion of certain important metals, such as zinc (Arts et al., 2018). It has been used to ward off lead, plutonium (De Bruin, 1967; Deblonde et al., 2018), and also iron poisoning (McDonald, 1966).

Ethylenediaminetetraacetic acid (EDTA) is a common iron chelator, which is a structural analog of DTPA. It is reduced by superoxide more rapidly than DTPA (Halliwell, 1978). EDTA chelates several metal ions, and its calcium disodium derivative was used in chelation therapy, such as for treating lead poisoning (Bjørklund et al., 2017). It was also used in the treatment of an iron overdose and cardiovascular disorders. However, some studies have suggested its poor efficiency (Matteucci et al., 2006; Lamas et al., 2013).

Other agents such as phytic acid (inositol hexaphosphate), 1,10-phenanthroline, and deferoxamine (desferrioxamine; DFO) are better inhibitors of iron-dependent hydroxyl radical generation than DTPA. Phytic acid has a history of use in the food industry as an AT. Its use has been discontinued in many countries because of its antinutrient properties, decreasing the absorption of various dietary metals (e.g., phosphorus, zinc, calcium, and copper) in the gastrointestinal tract (Bloot et al., 2023). On the other hand, phytic acid can provide a protective effect in the gut by sequestrating iron and preventing its pro-oxidant effect (Halliwell and Gutteridge, 2015).

1,10-Phenantroline readily chelates zinc, iron, and copper ions (Bencini and Lippolis, 2010). It was shown to prevent DNA degradation mediated by hydrogen peroxide (Mello-Filho and Meneghini, 1991). On the other hand, Cu^2+^–phenantroline complexes can lead to DNA damage (Bales et al., 2005).

DFO is a strong (but not entirely specific) chelator of Fe^3+^. It inhibits iron-dependent lipid peroxidation and conversion of hydrogen peroxide to hydroxyl radicals (Fenton’s reaction) in the presence of physiological buffer systems. DFO is a natural compound produced by *Streptomyces pilosus* that is of value in the prevention and treatment of acute iron poisoning, and in cases of repeated blood transfusion, e.g., in thalassemia, an inherited blood disorder characterized by decreased hemoglobin production. DFO seems to be safe, the daily doses can go up to 50 mg/kg body weight. However, there is a chance that patients will develop iron deficiency. DFO is not orally active and needs to be administered intravenously or subcutaneously (Merlot et al., 2013). Large doses can lead to hearing and visual disorders. These effects usually disappear on removal of the drug (Chen et al., 2005). A DFO overdose is also associated with certain bacterial and fungal infections, including those caused by *Yersinia enterocolitica*, *Vibrio vulnificus*, and *Rhizopus* spp. (Neupane and Kim, 2009). The Fe^3+^–DFO complex (known as feroxamine or ferrioxamine) is difficult to reduce, not only by superoxide but also other reducing agents (compared with Fe^3+^–DTPA). Despite having a high affinity for iron, DFO is unable to remove iron from important structures, such as transferrin. DFO is hydrophilic and therefore does not readily enter cell membranes (Halliwell and Gutteridge, 2015). It is sometimes part of organ preservation fluids, e.g., for heart transplantation (Chen et al., 2019).

**FIGURE 11:**
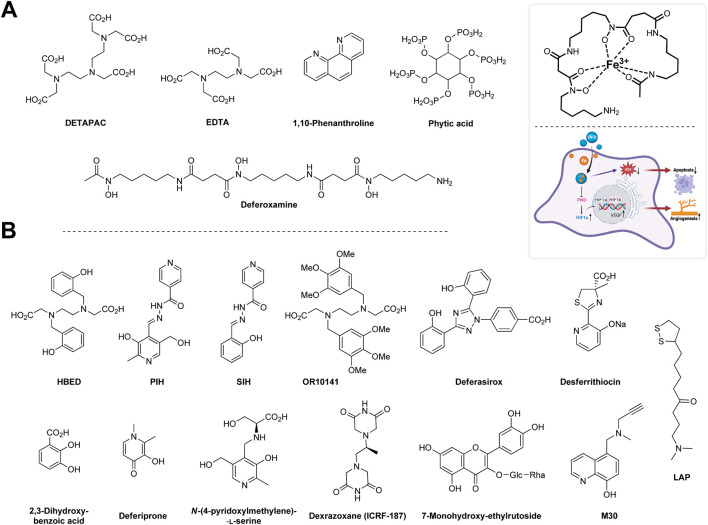
**(A)** Metal chelators and mechanism of action of DFO. DFO reduces free radical formation, diminishes apoptosis and induces hypoxia inducible factor (HIF) pathway and thus activates angiogenic pathways. **(B)** Other metal-chelating agents. Created with Biorender.com.

DFO was shown to scavenge various radicals, including superoxide, hydroxyl radical, and HOCl. It does not respond to peroxynitrite, although it can react with the RS derived from it, such as nitrogen dioxide and the carbonate radical. Again, the antioxidant action of DFO *in vivo* may be of little importance because the blood levels reached during therapy (∼20 μM) are too low to achieve the desired effect (Halliwell and Gutteridge, 2015). However, it may have other activities. DFO reduced chronic inflammation and symptoms of autoimmune diseases in animal studies (Di Paola et al., 2022). It also inhibited cell proliferation. Thus, the mechanism underlying the anti-inflammatory effect of DFO may be due to a reduction in the number of inflammatory cells (e.g., lymphocytes) (Choi et al., 2004). DFO also increases the levels of hypoxia-inducible factor (HIF-1) in various cell types (Figure 11A), resulting in amplified transcription of genes promoting erythropoiesis, glycolysis, and angiogenesis (Woo et al., 2006). The therapeutic benefits of DFO may be due to other non-AT activities. Increased HIF-1 activity is also observed for other iron chelators, such as salicylaldehyde isonicotinoyl hydrazone (SIH) and deferiprone (Creighton-Gutteridge and Tyrrell, 2002).

Among the DFO analogs are those where the DFO molecule has been attached to various high-molecular weight polymers, including cellulose, dextran, or hydroxyethyl starch. In these derivatives, the iron-binding activity has been significantly reduced. However, toxicity has also been decreased while the circulating plasma half-life has increased, and therefore, higher doses of these agents can be administered. They supposedly enter cells more readily than the original DFO molecule (Kalinowski and Richardson, 2005). These derivatives have been tested with various animal models of disease, e.g., septic shock and diabetes, where they showed a positive effect. DFO and its derivatives still have limited clinical use outside of its original indication, i.e., iron poisoning. They have been studied as a possible treatment for coronary artery disease (Duffy et al., 2001), spinal cord injury (Yao et al., 2019), and intracerebral hemorrhage (Zeng et al., 2018; Ren et al., 2020). Metal chelators discussed in this section are shown in Figure 11A.

### 10.1 Other metal-chelating agents

Hydroxypyridones were developed as orally active alternatives to DFO in the treatment of thalassemia. These include *N,N′*-bis(2-hydroxybenzyl)ethylenediamine-*N,N′*-diacetic acid (HBED), the hydrazones pyridoxal isonicotinoyl hydrazone (PIH) and salicylaldehyde isonicotinoyl hydrazone (SIH), desferrithiocin, and, 2,3-dihydrobenzoic acid. Some of these agents have been tested in animals and seem to be effective iron chelators. HBED was found to enhance iron elimination in monkeys (Bergeron et al., 1999). PIH demonstrated iron-chelating activity in hypertransfused rats and iron-loaded rat cardiomyocytes alone or in combination with DFO (Link et al., 2003). The structural analog SIH has only been studied in cell cultures, demonstrating protective effects against H_2_O_2_-induced damage in H9c2 cardiomyoblasts (Simunek et al., 2005). Various other structural derivatives of PIH and SIH have been synthesized, one of which is the compound 4,4′-((1E,1′E)-hydrazine-1,2-diylidenebis(ethan-1-yl-1-ylidene))bis(benzene-1,3-diol). This derivative exhibited relatively high inhibitory activity against ABTS and DPPH radicals (Tayade et al., 2022). 2,3-Dihydroxybenzoic acid was shown to chelate both copper and iron in various chelation models, such as the ferrozine assay (Harcárová et al., 2025). However, despite displaying promising results, some of them also have serious side effects, such as nephrotoxicity (Kalinowski and Richardson, 2005). This is also the case for desferrithiocin, which has been shown to be a potent iron chelator, but also demonstrated toxicity in animal studies (Nick et al., 2003). This prompted the development and investigation of safer derivatives. Much of the research has focused on 3-hydroxypyrid-4-ones (Halliwell and Gutteridge, 2015). One of these derivatives, deferiprone (3-hydroxy-1,2-dimethylpyridin-4(1*H*)-one; also known as L1), has been tested in humans and is clinically used in Europe to treat thalassemia. However, deferiprone also has serious toxicity, which led to it being withdrawn from clinical use in some countries. It seems to be more toxic than DFO (Kalinowski and Richardson, 2005). The most common side effects include coloration of urine (to a red-brownish color), nausea, abdominal pain, vomiting, and hepatic fibrosis (Brittenham et al., 2003; Hatcher et al., 2009). The toxicity of hydroxypyridones might be related to the Fe^3+^ complexes that may generate free radicals (Kalinowski and Richardson, 2005; Murakami and Yoshino, 2020) (compared with DTPA). Hydroxypyridones are able to release iron ions from biologically important structures (e.g., lactoferrin) (Halliwell and Gutteridge, 2015), potentially leading to the production of free radicals.

Another orally active iron chelator is deferasirox (Exjade^®^; ICL670), which has shown potential in clinical trials of thalassemia and other forms of chronic anemias and chronic iron overload (Cappellini, 2005; VanOrden and Hagemann, 2006). Deferasirox and deferiprone are the only oral medications approved for this purpose. Their use, however, is associated with some serious side effects, including kidney and liver failure, and gastrointestinal bleeding (Kontoghiorghes, 2013). Some compounds selectively chelate iron at the sites of oxidative stress and not elsewhere in the body. In the presence of hydrogen peroxide, they are converted to agents with increased chelation ability (e.g., hydroxylation of the aromatic ring), which may thus chelate iron more readily. An example of such a compound is OR10141. Research on this compound was carried out before 2004 (Galey et al., 2000). Another example is 1-(dimethylamino)-8-(1,2-dithiolan-3-yl)octan-4-one (LAP), a derivative of lipoamide that accumulates within lysosomes. It can protect a cell culture against H_2_O_2_-induced damage more effectively than DFO (Persson and Richardson, 2005). *N*-(4-Pyridoxylmethylene)-L-serine is a combination of serine and vitamin B_6_ (pyridoxamine). It protected the skin from UV light-induced damage in mice. Its activity may presumably be exerted by chelating iron (Kitazawa et al., 2005). The metal chelating ability has also been added to a few monoamine oxidase (MAO) inhibitors, such as M30, which was protective against MPTP-induced dopamine depletion in mice. It may be of potential value in the treatment of Parkinson’s disease (Gal et al., 2005). M30 has also decreased elevated levels of inflammatory cytokines in a mouse model of Alzheimer’s disease (Pimentel et al., 2015). It may also have therapeutic benefits with other diseases. For example, M30 protected rat hepatocytes against ethanol-induced injury (Xiao et al., 2015). Dexrazoxane (ICRF-187) is used clinically to attenuate the cardiotoxic effects of certain anticancer drugs (e.g., doxorubicin). Its cardiotoxicity-diminishing activity seems to be due to the removal of iron from iron–anthracycline complexes, and thus it may reduce free radical formation. Dexrazoxane also inhibits topoisomerase II activity (Deng et al., 2015). Dexrazoxane is structurally related to stilbenoids. 7-Monohydroxy-ethylrutoside seems to provide similar cardiotoxicity protection as dexrazoxane in mice (Jacobs et al., 2011). All agents discussed in this section are shown in Figure 11B.

## 11 RS generation inhibitors

### 11.1 Xanthine oxidase (XO) inhibitors

**FIGURE 12:**
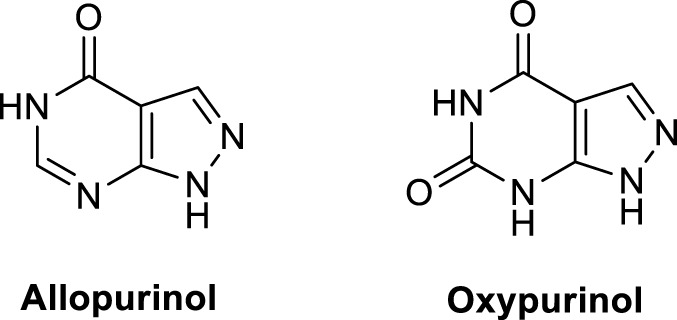
XO inhibitors based on a purine skeleton.

XO inhibitors are compounds that can inhibit XO activity, an enzyme responsible for the biosynthesis of purines. Inhibition of XO activity generally leads to reduced production of uric acid (which itself is an AT). Several XO-inhibiting drugs are indicated for treatment of hyperuricemia and related disorders, such as gout, a painful condition characterized by deposition of monosodium urate monohydrate crystals in joints (Dewick, 2009). XO inhibitors are also being studied as a possible treatment for reperfusion injury. XO inhibitors can be classified into two major groups: purine and non-purine analogs. The best-known purine XO inhibitor is allopurinol (Figure 12). It is clinically used to treat hyperuricemia and is sometimes added to organ preservation solutions. Allopurinol was also tested in many animal models of hypoxia-reoxygenation injury, where it showed positive effects. Since it has a long history of clinical use, its side effects are well known (Pacher et al., 2006). Generally, XO only contributes to RS generation to a limited extent in most human tissues (apart from the gastrointestinal tract). However, XO-derived RS production is increased in tissues affected by certain diseases, including atherosclerosis (Nasi et al., 2021), rheumatoid arthritis (Hanachi et al., 2009), or Dupuytren’s contracture (Murrell and Hooper, 1992). Whether administration of allopurinol in these conditions would provide human benefit is still unknown.

Oxypurinol (Figure 12) is a major metabolite of allopurinol, and it shares similar pharmacological activity (Pacher et al., 2006). In addition to XO inhibition, both compounds also have antioxidant activity and were shown to react with several RS *in vitro*, including HOCl (Augustin et al., 1994) and hydroxyl radicals (Moorhouse et al., 1987). However, the concentrations of both allopurinol and oxypurinol achieved during *in vivo* pharmacotherapy seem to be too low for their antioxidant effect (if there is any) to be of therapeutic importance (Halliwell and Gutteridge, 2015). XO inhibitors, including allopurinol and oxypurinol, are generally able to prevent oxidation of hypoxanthine. During hypoxia-reoxygenation injury, hypoxanthine is reincorporated into adenine nucleotides. This could be the mechanism by which XO inhibitors provide protective effects in ischemia/reperfusion-related conditions (Khatib et al., 2001). Various flavonoids, such as myricetin, kaempferol, quercetin, and inositols (e.g., phytic acid) have been found to inhibit XO *in vitro* and/or in model animals (Pacher et al., 2006). Again however, there is little evidence that these compounds would provide therapeutic benefits with conditions related to XO-derived RS production. These compounds are mentioned elsewhere in this text (e.g., chain-breaking ATs and metal chelators; Section 6.1 and 10, respectively).

### 11.2 Inhibitors of phagocyte RS production

**FIGURE 13:**
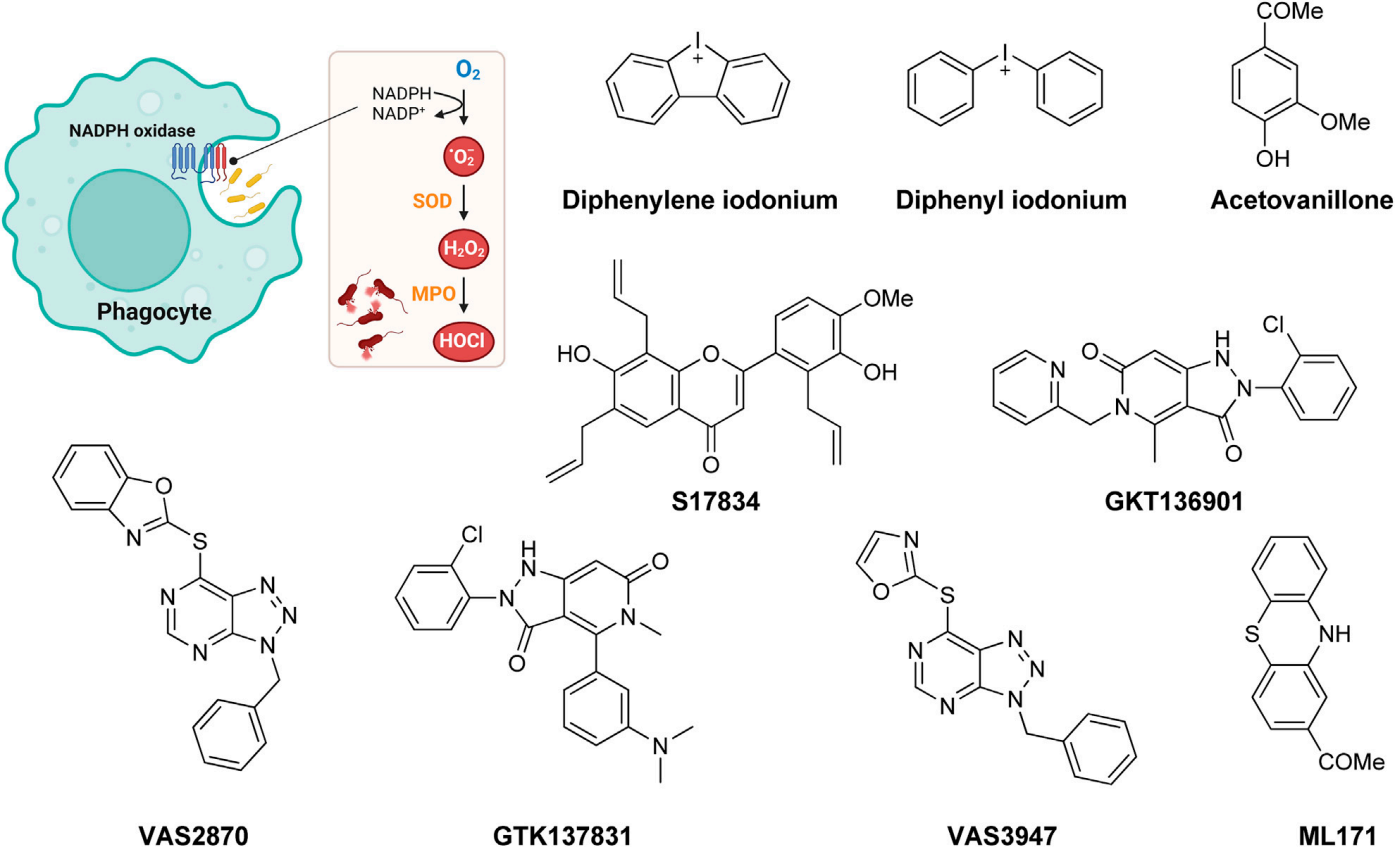
Suggested inhibitors of phagocyte-derived RS production and the important mechanism of respiratory burst for killing pathogens in phagocytes. Created with Biorender.com.

Phagocytes (such as neutrophils and macrophages) have an important role as the first line of defense of an organism against pathogens. They produce various RS, and this is one of the mechanisms by which they fight infection and provide subsequent immunity (Figure 13) (Dupré-Crochet et al., 2013). Insufficient production of RS by phagocytes can lead to serious complications. For example, patients with chronic granulomatous disease, caused by inherited defects in the phagocyte NADPH oxidase responsible for generating superoxide, are at increased risk of infections because they cannot effectively eliminate pathogens (Halliwell, 2012). On the other hand, excessive RS production can also lead to disorders, most commonly prolonged inflammation and related tissue injury (Mittal et al., 2014). Inhibitors of phagocyte recruitment may, therefore, be of greater value. These may include inhibitors of production/antagonists of the action of pro-inflammatory cytokines (e.g., infliximab) (Maini, 2004), compounds that decrease the production of adhesion molecules (e.g., β-amyrin) (Shih and Cherng, 2014), antibodies targeting adhesion molecules (Siles et al., 2018), or compounds that compete with phagocytes to bind the endothelium (Panés et al., 1999). Agents preventing normal RS production by phagocytes may also be of interest (Halliwell and Gutteridge, 2015).

Various anti-inflammatory drugs have been suggested to decrease phagocyte-derived superoxide formation. For example, some of the therapeutic benefits observed for ebselen could be associated with this activity as it also inhibits NADPH oxidase activity. Diphenylene iodonium (DPI) and structurally related analogs (e.g., diphenyl iodonium; DIP) inhibited respiratory burst in isolated neutrophils, but are nonspecific and are able to inhibit several other flavoproteins, including NOS, cytochrome P450 reductase, XO, and mitochondrial NADH-CoQ oxidoreductase (Altenhöfer et al., 2015; Cipriano et al., 2023). In addition, DPI seems to inhibit platelet aggregation (Stef et al., 2007). There have been negative effects observed as well, suggesting that DPI (and other related structures) act as pro-oxidants by decreasing GSH (Riganti et al., 2004). Acetovanillone (apocynin) is a natural 4-hydroxybenzoic acid derivative found in the roots of *Apocynum cannabinum* (Apocynaceae) and *Picrorhiza kurroa* (Plantaginaceae). Phenolic acids are found in nearly every higher plant. However, the distribution of acetovanillone is limited compared to other phenolic acids. Acetovanillone is oxidized by the myeloperoxidase enzyme to a product that blocks the assembly of NADPH oxidase. Thus, it only affects those activated phagocytes that are producing the hydrogen peroxide necessary for peroxidase activity (Altenhöfer et al., 2015). It showed promising anti-inflammatory activity in rats. However, high blood levels are required to achieve this effect (Simons et al., 1990; Boshtam et al., 2021). Similar to DPI and DIP, acetovanillone also exhibited reactive species–producing activity (Riganti et al., 2006).

Agents that can selectively inhibit one or more subunits of NADPH oxidase (e.g., NOX-1, NOX-2, NOX-3, NOX-4, and NOX-5) have been developed. Compounds mentioned above are nonspecific NADPH oxidase inhibitors and are not able to discriminate between different isoforms of NOX (with the exception of ebselen, which was later shown to hinder the activity of NOX-1, NOX-2, and NOX-5 at lower concentrations than its glutathione peroxidase mimetic activity; compare with GPx peroxidase mimetics in Section 5) (Altenhöfer et al., 2015; Cipriano et al., 2023).

S17834 is a flavonoid derivative that was developed by Servier Pharmaceuticals LLC. NADPH oxidase inhibition of S17834 was suggested from the observation that it prevented superoxide formation in human umbilical vein endothelial cells (HUVECs). The specific NOX isoforms inhibited by S17834, as well as its mode of action, remain unknown. However, HUVEC only expresses NOX-2 and NOX-4, suggesting that S17834 selectively inhibits these isoforms. It showed anti-atherosclerotic and anti-diabetic activity in various animal studies (Cayatte et al., 2001; Zang et al., 2006; Xu et al., 2011; Qin et al., 2012). S17834 can also activate adenosine monophosphate-activated protein kinase, so its therapeutic benefit may relate to this activity rather than NOX inhibition. However, more recent studies are limited (Altenhöfer et al., 2015; Cipriano et al., 2023).

GKT136901 and GKT137831 can inhibit NOX-1, NOX-4, and NOX-5 activities. The mechanism by which they exert their activity remains unknown. Since GKT136901 and GKT137831 are structurally related to NADPH, it seems they compete with NADPH at the active site of the NADPH oxidase. However, GKT136901 was found to scavenge peroxynitrite, which may complicate the interpretation of results from NOX-inhibitory assays (GKT137831 does not scavenge RS). GKT137831 has advanced to clinical testing, e.g., for diabetic nephropathy and idiopathic pulmonary fibrosis. It seems that its pharmacological properties are more suitable for clinical use compared to GKT136901 (Altenhöfer et al., 2015; Diebold et al., 2015; Cipriano et al., 2023).

ML171 showed the most selective NOX-1 inhibition out of 16,000 other phenothiazines tested in a structural relationship study at the Scripps Research Institute. However, it also inhibits NOX-2, -3 and -4 and XO to some extent. Since ML171 is structurally related to some commonly used phenothiazine-derived anti-psychotic drugs (e.g., chlorpromazine), it may interfere with receptors for serotonin and adrenaline (Altenhöfer et al., 2015; Diebold et al., 2015; Cipriano et al., 2023).

VAS2870 and VAS3947 (named VAS after the Vasopharm GmbH that developed them) are NOX-2 selective inhibitors. VAS2870 is slightly stronger than VAS3947. However, they are not entirely NOX-specific: VAS2870 is also able to inhibit the activity of NOX-4 and NOX-5, while VAS3947 inhibits the activity of NOX-1, NOX-2, and NOX-4. The mode of NOX-2 inhibition seems to stem from an inhibition of platelet-derived growth factor (PDGF)-mediated NAD(P)oxidase activation (Ten Freyhaus et al., 2006). Both agents have poor solubility, which may limit their use in clinical practice (Altenhöfer et al., 2015; Cipriano et al., 2023).

There are many other potential selective NOX inhibitors (e.g., fulvene-5, triphenylmethane derivatives, celastrol, some synthetic diarylheptanoids, grindelic acid, dietary flavonoids, shionogi I and II, and perhexiline). However, they often show direct RS scavenging or other biological activity (e.g., inhibition of topoisomerase II). The use of their NOX-inhibitory properties *in vivo* may thus be problematic (Altenhöfer et al., 2015; Casas et al., 2020; Cipriano et al., 2023). Structures of NOX inhibitors are shown in Figure 13.

## 12 Concluding remarks

ATs are of interest both as therapeutic agents and as food preservatives. Natural products, as well as semi-synthetic and synthetic analogs, are being actively investigated—based on the premise that structural modifications may yield compounds with superior properties compared to their natural counterparts. The proposed mechanisms of action and therapeutic benefits of synthetic ATs are summarized in Table 4. Despite their prevalence in the human diet, natural antioxidants, particularly dietary flavonoids, have largely failed to demonstrate therapeutic efficacy and may not serve as suitable templates for drug development. Although a wide range of semi-synthetic and synthetic derivatives have been developed and studied, the discovery of a medicinally effective antioxidant remains elusive. There is currently little conclusive evidence that oxidative stress-related diseases can be effectively treated by administering antioxidants. Many compounds have failed during preclinical or clinical evaluation. In cases where therapeutic benefits were observed in clinical trials, it often became apparent that these effects were unrelated to the compound’s antioxidant activity. For some agents, antioxidant activity may contribute to their efficacy, but therapeutic doses are frequently too low to elicit a meaningful antioxidant response. Additional limitations include poor solubility, low oral bioavailability, limited tissue distribution, and serious adverse effects. As a result, only a small subset of (semi-)synthetic antioxidant analogs appears to warrant further investigation. Among them, ebselen, along with other selenium- and tellurium-containing compounds, has shown some promise in both animal models and clinical trials targeting oxidative stress-related conditions. It is clinically used to treat stroke in some countries (Japan). More studies are, however, needed to prove its efficacy in other clinical scenarios and if its therapeutic benefits are indeed associated with antioxidant activity. Edaravone is used in the treatment of stroke and amyotrophic lateral sclerosis in some countries. However, it remains unclear whether it truly functions as an antioxidant–it is still not known whether it can scavenge free radicals at concentrations achievable in the body after a normal dose. No clinical study has measured oxidative stress levels following its administration, and its exact mechanism of action remains unknown. NAC is of value in the treatment of paracetamol overdose and is also used as a mucolytic agent. In the literature, NAC is often, albeit somewhat exaggeratedly, referred to almost synonymously with the term antioxidant. However, this compound also exhibits a range of other biological activities. As with edaravone and ebselen, it is therefore questionable whether its therapeutic benefits are primarily due to its antioxidant properties. Its ability to mitigate paracetamol toxicity is likely related to its role as a cysteine substitute in the synthesis of GSH. Paradoxically, this same mechanism may underlie its potential pro-cancer effects: by boosting GSH production, cancer cells can better protect themselves from oxidative damage caused by ROS (Ramachandran and Jaeschke, 2021). Mitochondria-targeted ATs (e.g., mitoquinol) have also shown potential. Additionally, various metal chelators are already used clinically to treat specific conditions. However, their therapeutic index is often narrow—as exemplified by deferoxamine, which, despite being well-studied, is currently limited to treating acute iron poisoning and conditions involving chronic blood transfusions (e.g., thalassemia). Enzymes of the NOX family have emerged as promising targets for antioxidant therapy. Compounds that selectively inhibit specific NOX isoforms, such as S17834 or GKT137831, may be particularly effective. Ebselen and structurally related compounds may exert their therapeutic effects, at least in part, through NOX inhibition. While NOX inhibitors hold significant promise, they are relatively new to antioxidant research, and additional studies are necessary to validate their clinical efficacy. In contrast, antioxidant-based food preservatives appear to be more successful in practical application. Nevertheless, safety concerns remain as some compounds—such as BHT and BHA—have been associated with potential toxicity. However, the concentration used in food is typically so low that toxic effects are considered unlikely. Still, several preservatives, such as nordihydroguaiaretic acid, have been withdrawn due to safety concerns.

**TABLE 4:** Mechanism of action and level of observed therapeutic benefits of semi-synthetic and synthetic AT mentioned in this review.

Compound	Mechanism of antioxidant action	Level of evidence	Comments	References
*SOD/catalase mimetics*	Neutralization of O_2_^•−^ and/or H_2_O_2_		SOD/catalase mimetics are suggested to undergo redox reactions and enter cells more readily than natural SOD/catalase enzymes	Day (2008), Halliwell and Gutteridge (2015)
SC-52608	Will react selectively with O_2_^•−^	Therapeutic benefit only observed in animal studies (cardiovascular, lung, CNS and liver, renal, and gastrointestinal models of disease), none of these compounds have proceeded to clinical trials		
SC-54417				
SC-55858				
M40401				
M40403				
Compounds of EUK series	Non-selective SOD/catalase mimetics that apart from O_2_^•−^ will also react with other RS			
FeTMPyP				
MnTMPyP				
Compounds of AEOL series				
*Spintraps*	Reaction with RS (O_2_^•−^) to form a nitroxide whose stability is significantly greater than that of the parent free radical			
PBN		Reported to provide protection of the CNS of animal models against free radical damage (e.g., rodent glioma models)	Is also able to release NO; activity may not be related to antioxidant action	Day (2008)
CPI-1429		Animal models (mice life span models)		Floyd et al. (2002)
NXY-059 (Cerovive^®^)		Advanced into human clinical trial with stroke where it failed to produce reproducible results	Poor antioxidant effect *in vitro*; activity may not be related to antioxidant action	Maples et al. (2004)
LPBNAH		Life span model (using *Philodina acuticornis*) and animal models (protection against ROS on isolated perfused rat hearts)		Tanguy et al. (2006)
STAZN		Animal model (reduced the severity of ischemia/reperfusion injury in a rat model)		Ley et al. (2008)
***Nitroxides***	Redox reaction with RS (O_2_^•−^)		Nitroxides show a range of redox reactions, not only those with superoxide; activity may not be related to antioxidant action	
TEMPO		Animal models (e.g., improved neurological function in a rat model of Huntington’s disease)		Yonekuta et al. (2007), Sandhir et al. (2015)
OXANO		Animal models (reduced the severity of ischemia/reperfusion injury in isolated rabbit lung)		Hasaniya et al. (2011)
3-nitratomethyl-PROXYL		No data available		
Hydroxamates		Animal models (reduced the severity of reperfusion injury in an isolated rat heart model)		Collis et al. (1993)
IAC		Animal models (e.g., improved the outcome in a rat model of colitis)		Vasina et al. (2009)
*GPx mimetics*	Elimination of H_2_O_2_			
Ebselen	Apart from GPx-mimetic activity, it also inhibits various isoforms of NADPH oxidase (NOX-1, -2, and -5)	Modest results from human clinical trials of stroke (phase II)	Also showed activities related to the anti-inflammatory effect; activity may not be related to antioxidant action	Yamaguchi et al. (1998), Parnham and Sies (2013)
BXT-51072		Promising improvement in patients with mild-to-moderate ulcerative colitis (phase II)	Severalfold more reactive in H_2_O_2_-eliminating activity than ebselen	(May 2016)
*Vitamin E derivatives*	Blocking autoxidation of organic molecules by quenching ROO^•^ radicals and forming stable radicals that do not propagate the oxidative chain			
BO-653		Anti-atherogenic effect (LDL-lowering ability) in a rabbit and mice models	Contain benzofuran ring instead of the chromane ring typical for vitamin E	Valgimigli and Amorati (2019)
IRFI005		Protected LDL from oxidation in an *in vitro* model		Barzegar et al. (2011)
Raxofelast		Inhibited lipid peroxidation in a mice model of burn wounds		Bitto et al. (2007)
Trolox		Various animal models (e.g., reduced 3-nitropropionic acid-induced neurotoxicity in rats)	Used in many *in vitro* antioxidant assays as a positive control	Halliwell and Gutteridge (2015)
MDL 74,405		Reduced hydroxyl radicals in stunned myocardium of dogs	Contains a quaternary ammonium group and thus is cardioselective	Kuo et al. (1995)
Troglitazone		Clinically used as an antidiabetic (due to blood sugar-lowering ability) and anti-inflammatory drug. However, due to hepatotoxicity, it has been withdrawn from use in some countries	Oxidizes to several radicals, including quinones	Kassahun et al. (2001)
CR-6		Animal models (brain ischemia/reperfusion injury in rats)		Halliwell and Gutteridge (2015)
CX-659S		Anti-inflammatory activity in animal models of contact hypersensitivity		Inoue et al. (2003)
ETS-GS		Anti-inflammatory activity in a rat model of colitis	Molecule combination of vitamin E, taurine, and GSH	Sugita et al. (2013)
*Vitamin C derivatives*	Apart from chain-breaking activity, they may provide a regeneration system for vitamin E			
Ascorbyl palmitate		Anticancer activity in a mice model of carcinoma	History of use in the food industry as preservatives	El -Far et al. (2022)
2-Octadecyl ascorbate		No data available		
EPC-K1		Animal models (e.g., renal ischemia/reperfusion injury in a rat model)	Combined phosphate ester of vitamin C and E	Yamamoto et al. (2011)
*Other chain-breaking AT*				
Probucol		LDL-lowering ability in patients with acute coronary syndrome (phase I)	History of use as a cholesterol-lowering agent; withdrawn from use due to its HDL-lowering activity	El-Demerdash et al. (2003), Guo et al. (2015)
Succinobucol (AGI-1067)		Failed in clinical phase III as an anti-atherosclerotic agent	Probucol derivative in which the HDL-lowering ability was significantly decreased	Muldrew and Franks (2009)
Idebenone		Tested in clinical trials for Alzheimer’s disease and Friedreich’s ataxia, but approval was withdrawn due to the lack of demonstrated efficacy. Showed positive outcomes in a phase III trial for Leber’s hereditary optic neuropathy	A coenzyme Q derivative	Gutzmann et al. (2002)
Vatiquinone (EPI-743)		Currently undergoing phase III clinical trials for Friedreich’s ataxia	A coenzyme Q derivative. Inhibitor of 15-lipoxygenase	Zesiewicz et al. (2018)
OPC-14117		Demonstrated a protective effect in animal models (e.g., prevented neuronal death in a mouse model of neurodegenerative disease), but did not show therapeutic benefit in patients with Huntington’s disease		Abe et al. (1997), Dickey and La Spada (2018)
BN-82451		Showed neuroprotective effects in animal models of cerebral ischemia, Parkinson disease, Huntington disease, and amyotrophic lateral sclerosis	The benefit may stem from COX inhibition	Chabrier and Auguet (2007)
LY-178002		Provided some benefit in animal models of rheumatoid arthritis and cerebral ischemia/reperfusion		Halliwell and Gutteridge (2015)
LY-256548				
ONO-3144		Showed anti-inflammatory activity in animal models	The anti-inflammatory action may be caused by COX inhibition	
MK-477				
Promethazine		Inhibits lipid peroxidation in a model involving exposure of rat liver fractions to CCl4	Clinically used in treatment of allergies and may secondarily provide antioxidant action	Poli et al. (1989)
Chlorpromazine		Was shown to inhibit lipid peroxidation in liposomes and microsomes	Clinically used in treatment of psychiatric disorders and may secondarily provide antioxidant action	Eluashvili et al. (1978), Bindoli et al. (1988)
DPPD		Reduced atherosclerosis in ApoE^−/−^ mice (presumably via inhibition of lipid peroxidation)	Used as an antioxidant in lubricant and polymer industries	Tangirala et al. (1995)
Ethoxyquin		History of use as an antioxidant in the food industry	Was withdrawn from use due to mutagenic activity	Błaszczyk et al. (2013)
HDC		*In vitro* lipid-peroxidation inhibitory activity		Dandona et al. (2007)
Carvedilol		Reduced infarction size in a rabbit model of ischemia/reperfusion	Used as an antihypertensive drug (due to β-blocking activity)	
3-Hydroxycarvedilol			Some degree of evidence that it can decrease products of lipid peroxidation in humans	Malig et al. (2016)
SUN-N8075		Protective activity in light-induced retinal damage in rats and neuroprotective effect in mice models of amyotrophic lateral sclerosis and Huntington’s disease	Also shown to block Na^+^ and Ca^2+^ channels	Tanaka et al. (2010), Noda et al. (2013), Ojino et al. (2014)
BHA		Used as an AT in the food industry	Potentially carcinogenic, however at levels several times higher than those used in food	Shaw (2018)
BHT				
TBHQ				
Propofol		Inhibition of lipid peroxidation in isolated rat liver mitochondria	Clinically used as an anesthetic drug; may secondarily also provide antioxidant action at levels similar to those used during anesthesia	Eriksson et al. (1992), Han et al. (2022)
Propyl gallate		Used as an antioxidant in the food industry (oils and fats)	May also act as an LOX inhibitor and bind iron ions	Binbuga et al. (2005), Georgieva et al. (2013)
Nordihydroguaiaretic acid		History of use as an antioxidant in the food industry	Was withdrawn from use due to renal and hepatic toxicity	Manda et al. (2020)
Daflon		Used clinically in treatment of chronic venous insufficiency	Mechanism of action may not be related to the antioxidant activity	Lyseng-Williamson and Perry (2003)
Silybin bishemisuccunate		Clinically used as an antidote (e.g., mushroom poisoning)	Apart from the antioxidant activity, it may also inhibit absorption of toxins	Tauchen et al. (2020)
*Lazaroids*	Commonly used drugs conjugated with antioxidant molecules			
Tirilazad mesylate		Several clinical trials of stroke and traumatic brain injury where it failed to provide therapeutic benefits	Seems to be metabolized more rapidly in women than men	Cahill and Hall (2017)
U-101033E		Attenuated post-ischemic loss of dopaminergic nigrostriatal neurons in gerbils	They cross the blood–brain barrier more readily then tirilazad	Andrus et al. (1997)
U-104067F				
Edaravone	May act as an anti-inflammatory drug rather than an antioxidant	Clinically used to treat stroke and amyotrophic lateral sclerosis		Halliwell (2024)
*Thiols*	Direct scavenging of RS			
Glutathione		Clinical trials: e.g., it lowered 8-OHdG in diabetic patients but had no effect on fecal inflammatory markers in cystic fibrosis (both phase I)	May have serious side effects, e.g., bronchoconstriction in asthma patients	Bozic et al. (2020), Kalamkar et al. (2022)
OTC		Reduced oxidative stress (levels of ROS and inflammation markers) in HIV patients (phase I/II)		Halliwell and Gutteridge (2015)
*N*-Acetylcysteine (NAC)	Will also increase glutathione levels	Clinically used, e.g., to treat paracetamol overdose	Have shown other non-antioxidant activities as well, including interaction with NMDA and AMPA receptors and inhibition of NF-κB activity	Murphy et al. (2011), Forman and Zhang (2021)
*N*-Acetylcysteine amide		Reduced inflammatory markers and lipid peroxidation in various animal models of disease (e.g., multiple sclerosis, Parkinson’s disease, and cataract)	Was reported to cross the blood–brain barrier more readily than NAC	Sunitha et al. (2013)
*Other thiols*	Usually act by restoring glutathione levels		Many thiols are used in organ preservation solutions, where they diminish RS	
Mercaptoethylguanidine		Anti-inflammatory effect in a rat model of carrageenan-induced paw edema	Therapeutic activity may not directly relate to the antioxidant effect	Cuzzocrea et al. (1998)
Lipoic acid		Clinically used in some countries to treat diabetic neuropathy		Javed et al. (2015)
Bucillamine		Clinically used in Asia as an antirheumatic drug	Apart from anti-inflammatory properties, may also provide antioxidant action through thiol donation (is 16-fold more efficient in restoring GSH than NAC)	Horwitz (2003)
Cysteamine		Clinically used to treat cystinosis	Therapeutic activity may not directly relate to the antioxidant action	de Matos et al. (1995)
Dimercaprol	They may also act as a metal chelator	Clinically used to treat arsenic poisoning		Mückter et al. (1997)
Penicillamine		Clinically used to treat arsenic and copper poisoning		Cui et al. (2005)
Mesna		Clinically used as a chemotherapy adjuvant drug		Pendyala et al. (2000)
Mercapropionylglycine		Clinically used to treat cystinuria		Bartekova et al. (2018)
Amifostine		Clinically used as a radiotherapy protectant		Nair et al. (2001)
*Mitochondria-targeted AT*	Molecules specifically designed to enter the mitochondria and retard the mitochondria-associated RS production			
Mitoquinol		Used in phase II trials of Parkinson disease and chronic hepatitis C	Seems to cross the blood–brain barrier	Halliwell and Gutteridge (2015)
*Metal chelators*	Mainly chelate various metal ions, especially iron, however, may also scavenge various RS			
DTPA (DETAPAC)		Clinically used as part of chelation therapy (not so much anymore due to depletion of Zn and Mg)	Fe^2+^–DTPA complexes can produce RS (e.g., OH^•^)	Arts et al. (2018)
EDTA		Chelates several metals, including Fe, Cu and Mg. EDTA infusion has been used in some countries as a part of chelation therapy in the treatment of vascular diseases, but its value is uncertain	It is reduced by O_2_^•−^ more rapidly than DTPA	Lamas et al. (2013)
1,10-Phenanthroline		Prevented H2O2-mediated damage to DNA in mammalian cell lines	Cu2+–phenantroline complex can cause DNA damage	Bencini and Lippolis (2010)
Phytic acid		Used in the food industry as an antioxidant, but withdrawn in some countries due to cases of dietary mineral malabsorption		Bloot et al. (2023)
Deferoxamine (DFO)	Can scavenge various RS, however, at doses not achieved during therapy	Clinically used (intravenously) in treatment of iron poisoning and chronic anemias	It also shows other biological activities as well (e.g., anti-inflammatory); therapeutic benefit may not be entirely associated to the antioxidant activity	Yao et al. (2019)
HBED		Was shown to induce net Fe excretion in monkeys		Bergeron et al. (1999)
PIH		Showed Fe chelating potential in hyper-transfused rats and in Fe-loaded rat heart cells		Link et al. (2003), Kalinowski and Richardson (2005), Simunek et al. (2005), Merlot et al. (2013)
SIH		Protected H9c2 cardiomyoblast cells against H2O2-mediated damage		
Desferrithiocin		Powerful Fe chelator; it has proven to be toxic in animals, leading to research into safer analogs		Nick et al. (2003)
2,3-Dihydroxybenzoic acid		Was shown to chelate Cu and Fe in different chelation models (e.g., ferrozine method)	Orally active	Harcárová et al. (2025)
Deferiprone (L1)		Clinically used to treat thalassemia, however, not used in some countries due to toxicity (e.g., hepatic fibrosis)	Orally active	Brittenham et al. (2003)
Deferasirox (Exjade^®^)		Clinically used in treatment of iron poisoning and chronic anemias		Cappellini (2005)
OR10141		Protected DNA against RS-induced single strand breaks in an *in vitro* model	Oxidative activation by H2O2 yields molecules with better Fe chelating ability	Galey et al. (2000)
LAP		Prevented lysosomal rupture and apoptotic cell death in cell cultures exposed to H2O2	Designed to accumulate in lysosomes	Persson and Richardson (2005)
M30		Showed protective effects against MPTP-induced dopamine depletion in mice	Also has MAO inhibition	Pimentel et al. (2015)
*N*-(4-Pyridoxylmethylene)-L-serine		Chelates Fe. Also showed protective effects against skin damage in hairless mice irradiated with UVB	Molecule combines serine and vitamin B_6_	Kitazawa et al. (2005)
Dexrazoxane (ICRF-187)		Clinically used to attenuate cardiotoxic effects of anticancer drugs (e.g., doxorubicin)	It presumably acts by removing iron from Fe^2+^-doxorubicin complexes; however, it also inhibits topoisomerase II activity	Deng et al. (2015)
7-Monohydroxy-ethylrutoside		Provides similar cardioprotective activity as dexrazoxane in animal models		Jacobs et al. (2011)
*Xanthine oxidase inhibitors*	Decrease RS production by inhibition of xanthine oxidase			
Allopurinol		Clinically used to treat hyperuricemia and added to organ preservation solutions	Apart from the XO-inhibiting activity, they also react with several RS *in vitro*, including HOCl and OH^•^	Pacher et al. (2006)
Oxypurinol		Major metabolite of allopurinol		
*RS-generation inhibitors*	Decrease RS production by inhibition of NADPH oxidase			
Diphenylene iodonium	Non-selective NADPH oxidase inhibitors	Inhibited NADPH oxidase activity (ROS production) in isolated neutrophils	Also shown to inhibit NOS, cytochrome P450 reductase, XO, and mitochondrial NADH-CoQ oxidoreductase; may produce RS	Altenhöfer et al. (2015), Cipriano et al. (2023)
Diphenyl iodonium				
Acetvanillone		Protected rats from acetic acid-induced colonic inflammation	May produce RS	
S17834	Selective inhibition of NOX-2 and -4	Inhibited the production of TNF-α in endothelial cells		
GKT136901	Selective inhibition of NOX-1, -4 and -5	Showed renoprotective effects in a mouse model of type 2 diabetes	Can also scavenge ONOO^−^	
GKT137831		Has entered human clinical trials of type 2 diabetes (results still not available)		
ML171	Selective inhibition of NOX-1	NOX-1 cellular inhibitory screening assay		
VAS2870	Selective inhibition of NOX-2	Has shown promising beneficial effects in preclinical disease models of thrombosis, neurodegeneration, and cancer value uncertain due to the lack of selectivity for individual NOX isoforms	Also inhibits NOX-4, and -5	
VAS3947			Also inhibits NOX-1, -2, and -4	

### 12.1 Future prospects

Countless ATs have been synthesized to date, and many more will undoubtedly follow. However, in order to truly discover an AT with meaningful therapeutic effects, a shift in the current testing paradigm is essential. A critical oversight in many studies is the assumption that oxidative stress is the primary, or sole, cause of the disease in question. In reality, oxidative stress may only be a secondary or downstream factor in many pathological processes. Therefore, it is important to distinguish between diseases in which oxidative damage plays a primary causal role and those in which it represents a late-stage consequence. Preclinical and clinical trials should be designed accordingly, with a focus on conditions where antioxidant therapy is more likely to yield therapeutic benefit. Among the most promising targets for such strategies are certain types of cancer, neurodegenerative diseases (e.g., Alzheimer’s disease and Parkinson’s disease), and chronic inflammatory disorders (Aliev et al., 2008; Halliwell, 2009).

Additionally, studies evaluating the role of ATs in human diseases rarely define which specific ROS are involved, their sources, how they reach their molecular targets, and which targets are affected, and why. Oxidative damage is often perceived as a random, nonspecific process, impacting a broad range of biomolecules. However, contrary to this common belief, ROS frequently exhibit selective reactivity. For example, increased oxidative damage to guanine in Parkinson’s disease or to specific proteins in Alzheimer’s disease suggests a high degree of specificity (Halliwell, 2006; Butterfield and Halliwell, 2019). The mechanisms underlying this selectivity remain largely unclear. Moreover, many studies fail to assess or report how administered antioxidants are distributed to the sites where they are expected to act, whether at the tissue level (e.g., brain) or subcellular level (e.g., mitochondria) (Cimmino et al., 2024). In some cases, administration of certain ATs may even result in the formation of harmful oxidative by-products, the biological effects and metabolic fate of which are poorly understood. While the tissue and cellular localization of endogenous antioxidants, such SODs, catalase, GPx, and peroxiredoxins, is relatively well characterized, equivalent data for most exogenous (natural or synthetic) antioxidants are lacking. Specifically, it remains unclear how their concentrations change within various tissues and organelles following administration. As a result, we cannot confidently determine whether such compounds effectively reduce ROS levels at relevant sites of oxidative stress. Additionally, many studies do not report changes in biomarkers of oxidative damage following AT treatment, making it difficult to assess whether any observed therapeutic benefit is actually attributable to the antioxidant activity. Future research should aim to provide a more detailed understanding of the mechanisms of action of candidate antioxidants, ideally at the molecular level (Halliwell, 2022).

In a similar vein, much of the available research based on animal models overlooks the inherent limitations of these systems and the potential for misleading results. Rodent models, in particular, raise concerns due to their limited translational relevance to human physiology. For instance, in rodent models of dementia and stroke, the administration of antioxidants has been shown to reduce oxidative stress and improve disease outcomes. However, these effects have not been consistently replicated in human clinical trials. One contributing factor may be the substantial differences in drug metabolism between rodents and humans. This discrepancy is not unique to antioxidants; it has also been observed with many anticancer agents that were effective in rodent models but failed to demonstrate efficacy in human trials. An additional confounding factor is that rodents, unlike humans, can synthesize their own vitamin C (Padayatty and Levine, 2016). As a result, positive outcomes in rodent studies may partly reflect an upregulation of endogenous antioxidant defenses rather than the effects of the administered antioxidant itself. Limitations are also evident in rodent models of neurodegenerative diseases, particularly those induced by toxins such as rotenone or MPTP to mimic Parkinson’s disease. In humans, Parkinson’s disease is rarely caused by acute toxic exposure; it is more commonly linked to a combination of environmental and genetic factors. Consequently, these toxin-based models do not fully recapitulate the human disease pathology and may therefore yield results that are not directly applicable to clinical settings (Potashkin et al., 2011). Models utilizing cell cultures and *in vitro* tests also have their limitations. Studies have shown that cell cultures can adapt to oxidative stress, for example, by increasing the excretion of pyruvate. Moreover, *in vitro* tests often rely on free radicals that do not naturally occur in biological systems (e.g., DPPH, AAPH, and ABTS), raising doubts about the relevance of their results to *in vivo* conditions (Murphy et al., 2011). In our view, the main research gap in current antioxidant testing lies in the insufficient awareness of these limitations and excessive reliance on such results. The closest model to human clinical settings (and with the highest clinical relevancy when humans are excluded) is that using primates; however, this is constrained by ethical and economic reasons.

Over the past decade, numerous novel technologies have emerged that may rekindle interest in antioxidant agents previously excluded from development or unsuccessful in earlier clinical trials. The reasons for these failures often include short *in vivo* half-life, chemical instability, limited cellular uptake, poor bioavailability, lack of targeted delivery to specific tissues or (sub)cellular compartments, a narrow therapeutic index, and toxicity. To overcome these challenges, a range of advanced strategies have been employed to modify either the antioxidant molecule itself or its delivery system. These include prodrug strategies, nanotechnology-based delivery systems, conjugation with different polymers (e.g., PEG), encapsulation with the use of metal–organic frameworks, targeted delivery using ligand conjugation (e.g., peptides and antibodies), or manufacture of inhalable devices (e.g., intranasal administration). These technological advancements may significantly improve the pharmacological profile of antioxidant agents and enhance their therapeutic potential in future clinical applications. A relatively recent approach involves the use of various molecules—such as calix[n]arenes, resorcinarenes, calixtyrosols, calixpyrroles, cucurbit[n]urils, and porphyrins—for the formation of crown-like macrocycles and supramolecular structures composed of two to eight units. These compounds have been shown to suppress a range of radicals, both non-biological (e.g., DPPH and ABTS) and biologically relevant (e.g., superoxide, peroxide, alkyl radicals, and H_2_O_2_). Some, such as calix[n]arenes, have demonstrated the ability to inhibit azobisisobutyronitrile-induced linoleic acid peroxidation, while others, like porphyrins, are effective in suppressing peroxidation processes *in vivo*. Moreover, these scaffolds—particularly calix[n]arenes and cucurbit[n]urils, can be loaded with various bioactive compounds (e.g., small molecules such as curcumin, resveratrol, or TEMPO and antioxidant enzymes such as catalase or glutathione peroxidase), which can either enhance their intrinsic antioxidant activity or facilitate their targeted delivery to specific cells (Lee et al., 2020; Maldonado-Sanabria et al., 2024). Targeting antioxidants specifically to the mitochondria is of particular interest, given that more than 80% of reactive oxygen species (ROS) are generated at this site. This approach aims to maximize the efficacy by directing antioxidants to the primary source of oxidative stress within cells. Alternative strategies have also been proposed. For example, antioxidant gene therapy has been suggested as a means of overcoming the problem of poor delivery and bioavailability of antioxidant compounds at their intended sites of action (Firuzi et al., 2011). Interestingly, pro-oxidants may also hold therapeutic potential in oxidative stress-related diseases. By inducing mild oxidative stress, they can activate endogenous defense systems, particularly the Nrf2 pathway, which plays a central role in regulating antioxidant responses. However, it is important to acknowledge that free radicals are not inherently harmful and serve essential functions in normal physiology, such as cell signaling and immune responses. Therefore, excessive, untargeted, or poorly controlled suppression of reactive species may disrupt physiological processes and result in serious adverse effects.

Many of the advanced technologies mentioned above have demonstrated promising results. However, numerous preclinical and clinical studies involving these innovations have failed to account for the fundamental limitations in current antioxidant testing approaches. Arguably, the most critical step toward progress is a paradigm shift in how antioxidants are evaluated. Without such a change, the development of functionally effective and clinically applicable antioxidant therapies will likely remain elusive.

## Abbreviations

8-OHdG, (8-hydroxy-2′-deoxyguanosine); ABTS, (2,2′-azino-bis(3-ethylbenzothiazoline-6-sulfonic acid); AD4, N-acetylcysteine amide; AMPA, α-amino-3-hydroxy-5-methyl-4-isoxazolepropionic acid receptor; AT, antioxidant; BBB, blood–brain barrier; CoQ, coenzyme Q; COX, cyclooxygenase; DFO, deferioxiamine (desferrioxamine); DPPH, 2,2-diphenyl-1-picrylhydrazyl radical; DIP, diphenyl iodonium; DPI, diphenyleneiodonium; DPPD, *N,N′*-diphenyl-p-phenylene diamine; DTPA, diethylenetriaminepentaacetic acid; EDTA, ethylenediaminetetraacetic acid; GPx, glutathione peroxidase; GSH, glutathione; HBED, *N,N′*-bis(2-hydroxybenzyl)ethylenediamine-*N,N′*-diacetic acid; HDC, 5,8-dimethyl-9*H*-carbazol-3-ol; HDL, high-density lipoprotein; HOCl, hypochlorous acid; IBD, inflammatory bowel disease; LOX, lipoxygenase; MAO, monoamine oxidase; MOF, metal–organic frameworks; MPO, myeloperoxidase; MPTP, 1-methyl-4-phenyl-1,2,3,6-tetrahydropyridine; NAC, *N*-acetylcysteine; NF-κB, nuclear factor kappa B; NMDA, N-methyl-D-aspartic acid; NO, nitric oxide; NOS, nitric oxide synthase; NOX, NADPH oxidase; Nrf2, nuclear factor erythroid 2-related factor 2; OCTN1, organic cation/carnitine transporter 1; ONOOH, peroxynitrous acid; OTC, 2-oxothiazolidine-4-carboxylic acid; PBN, α-phenyl-*tert*-butylnitrone; PIH, pyridoxal isonicotinoyl hydrazine; PLGA, poly(lactic-co-glycolic) acid; PUFA, polyunsaturated fatty acid; RNS, reactive nitrogen species; ROS, reactive oxygen species; RS, reactive species; SOD, superoxide dismutase; TBI, traumatic brain injury; TPP, triphenylphosphonium; XO, xanthine oxidase.
